# A Generalized Physiologically Based Kinetic Model for Fish for Environmental Risk Assessment of Pharmaceuticals

**DOI:** 10.1021/acs.est.1c08068

**Published:** 2022-04-26

**Authors:** Jiaqi Wang, Tom M. Nolte, Stewart F. Owen, Rémy Beaudouin, A. Jan Hendriks, Ad M.J. Ragas

**Affiliations:** † Department of Environmental Science, Radboud Institute for Biological and Environmental Sciences, Radboud University, Nijmegen 6500 GL, The Netherlands; ‡ AstraZeneca, Global Sustainability, Macclesfield, Cheshire SK10 2NA, United Kingdom; § Institut national de l’environnement industriel et des risques (INERIS), Verneuil-en-Halatte 60550, France; ∥ Department of Environmental Sciences, Faculty of Science, Open University, Heerlen 6419 AT, The Netherlands

**Keywords:** physiologically based kinetic model, fish, pharmaceuticals, internal concentrations, ionization

## Abstract

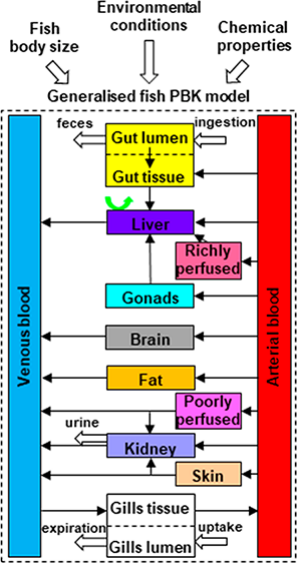

An increasing number of pharmaceuticals found in the environment potentially impose adverse effects on organisms such as fish. Physiologically based kinetic (PBK) models are essential risk assessment tools, allowing a mechanistic approach to understanding chemical effects within organisms. However, fish PBK models have been restricted to a few species, limiting the overall applicability given the countless species. Moreover, many pharmaceuticals are ionizable, and fish PBK models accounting for ionization are rare. Here, we developed a generalized PBK model, estimating required parameters as functions of fish and chemical properties. We assessed the model performance for five pharmaceuticals (covering neutral and ionic structures). With biotransformation half-lives (HLs) from EPI Suite, 73 and 41% of the time-course estimations were within a 10-fold and a 3-fold difference from measurements, respectively. The performance improved using experimental biotransformation HLs (87 and 59%, respectively). Estimations for ionizable substances were more accurate than any of the existing species-specific PBK models. The present study is the first to develop a generalized fish PBK model focusing on mechanism-based parameterization and explicitly accounting for ionization. Our generalized model facilitates its application across chemicals and species, improving efficiency for environmental risk assessment and supporting an animal-free toxicity testing paradigm.

## Abstract 2

A generalized fish PBK model was developed with mechanistic extrapolation and provided reasonable predictions, facilitating chemical risk assessments for fish.

## 1 Introduction

With increasing medical and veterinary use,^1^ pharmaceuticals have been detected as emerging pollutants in global water bodies.^2−4^ Many of these pharmaceuticals reside in aquatic organisms such as fish, potentially imposing adverse effects on organism survival or fitness and ecosystem health.^5−7^ Consequently, regulations have been established on marketing authorization and environmental risk assessment of pharmaceuticals (e.g., ref (8)).

Due to financial and ethical constraints and the increasing number of chemicals involved, it is impractical to test all pharmaceuticals’ effects on fish and other aquatic organisms.^9,10^ Physiologically based kinetic (PBK) models provide estimates of chemical concentrations over time in specific tissues based on descriptions of absorption, distribution, metabolism, and excretion processes and the physiology and anatomy of the organism.^11^ PBK models have become essential risk assessment tools since they allow a mechanistic framework to understand toxicological effects in organisms.^12,13^ To date, for environmental risk assessment, fish PBK models have been restricted to a few species (e.g., rainbow trout) where full sets of input parameters (preferably experimental values) were available.^11,14^ While these models are useful in species-specific risk assessments, risk assessors have to deal with countless species. Within this context and the aim to phase out animal testing,^15^ a more generalized PBK model is needed, applicable to a broader range of fish species and substances.^16^

For the generalized modeling approach, required input parameters can be estimated as (mechanistic) functions of fish and chemical attributes. Regarding physiological data (fish-related, e.g., tissue volumes), many variations in physiological processes can be explained by body mass (allometric scaling) and temperature (Boltzmann–Arrhenius kinetics).^17−19^ Regarding biochemical parameters (fish- and chemical-related, e.g., partitioning coefficients), previous studies have developed quantitative structure–activity relationships (QSARs),^20−23^ facilitating extrapolation to different chemicals. By estimating input parameters as functions of readily available biological and chemical properties, the PBK modeling process becomes less intensive in terms of data requirements. Additionally, the generalized model will facilitate its application across chemicals and species, increasing the domain of applicability. Within a risk assessment context, the generalized modeling approach can improve feasibility, efficiency, and transparency of exposure assessment, especially for species and substances for which empirical data are limited.

Unlike neutral chemicals, over 60% of the active pharmaceutical ingredients (APIs) bear a net charge at physiological pH.^24^ Given the wide range of pH in natural water bodies,^25^ the fate and toxicity of APIs could vary significantly in fish. For example, the bioconcentration factor (BCF) of diphenhydramine (acid dissociation constant (p*K*_a_) = 8.98) in fathead minnows increased from 4.2 L/kg at pH 6.7 to 53.3 L/kg at pH 8.7.^26^ The BCFs of fluoxetine (p*K*_a_ = 10.1) in Japanese medaka were 8.8 L/kg at pH 7 and 260 L/kg at pH 9.^27^ Fish PBK models are conventionally developed for neutral organic chemicals, rarely accounting for ionization.^28,29^

Hence, our study aimed to develop and evaluate a generalized fish PBK model to support environmental risk assessment of pharmaceuticals. To this end, the model was first parametrized by estimating required PBK inputs as functions of fish and chemical attributes to allow for a mechanistic interpretation and relieve intensive data requirements. The developed model was subsequently applied to five pharmaceuticals: carbamazepine (neutral), diclofenac (acid), ibuprofen (acid), diphenhydramine (base), and fluoxetine (base). Finally, we determined model performance by comparing estimated with measured concentrations in various tissues in fish.

## 2 Materials and Methods

### 2.1 PBK Model Structure and Implementation

**Figure 1:**
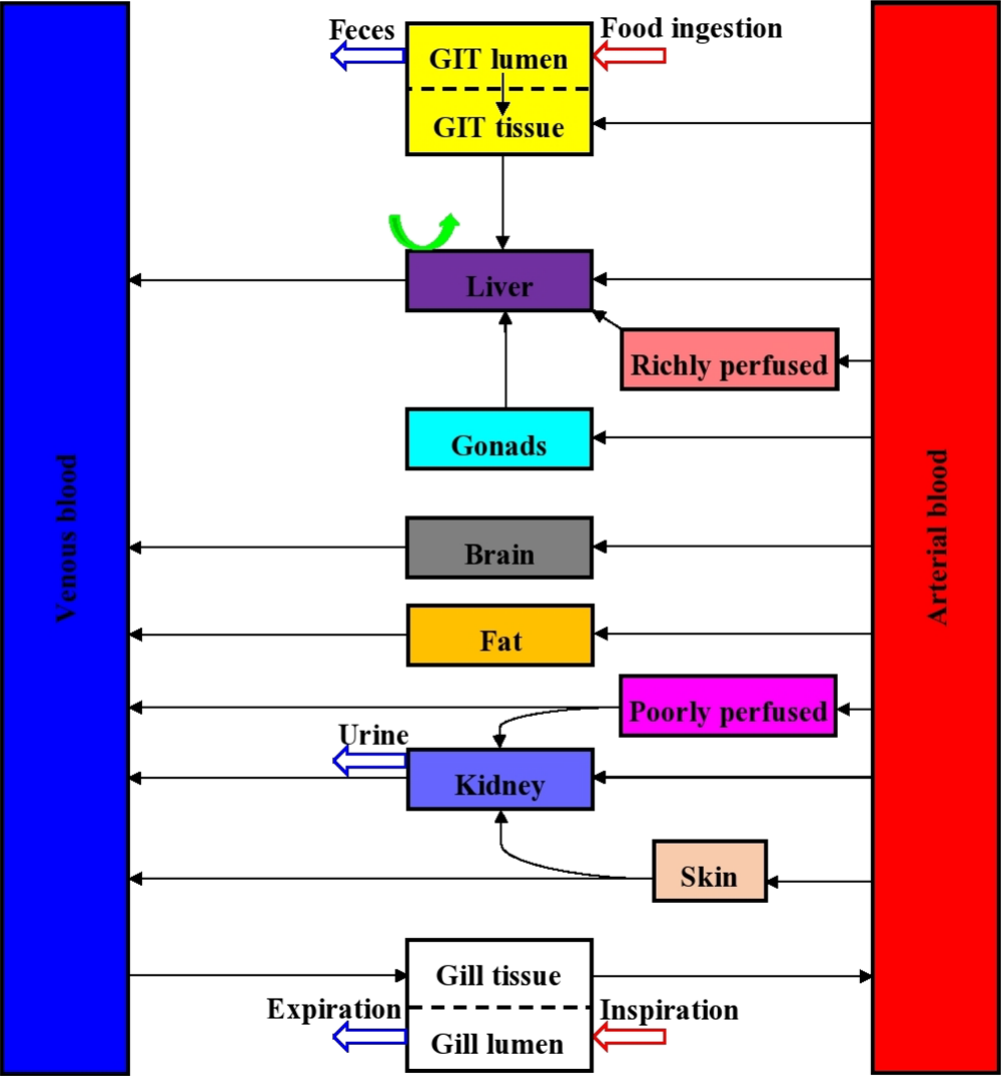
Scheme of the generalized fish PBK model. Boxes represent fish compartments. Black arrows represent blood flow. Red and blue arrows represent exposure and excretion routes in respective tissues, respectively. The green arrow represents hepatic metabolism.

The PBK model parameterized and applied in our study was based on a recent study^30^ (Figure 1). The model consists of 11 compartments: arterial and venous blood, gastrointestinal tract (GIT), skin, kidney, fat, liver, gonads, brain, poorly perfused tissues (PPT; skeleton and muscles), and richly perfused tissues (RPT; the remaining viscera including the heart, spleen, etc.). All compartments were assumed to be well-mixed with a blood flow-limited distribution. Absorption of a pharmaceutical can occur via the gills (water inspiration) and the GIT (food ingestion). Elimination can occur via urine, feces, expiration, and hepatic metabolism. Detailed model descriptions and equations are provided in the Supporting Information (SI, Section S1). The PBK model was coded using Excel with all equations solved by numerical integration^31^ and is available upon request.

### 2.2 Input Parameters

We derived input parameters as functions of fish and chemical attributes based on both mechanistic (e.g., allometric scaling with theoretical scaling exponents) and statistical (e.g., QSARs) relationships. We estimated (i) physiological parameters based on the fish body mass and temperature and (ii) biochemical parameters based on fish and chemical properties, taking pH influence into account (parameter summary in Table S1, SI). Consequently, only the fish body mass, the chemical properties, and the exposure scenario were required as inputs.

#### 2.2.1 Physiological Parameters

Physiological parameters include tissue volumes, tissue composition, cardiac output and oxygen consumption, and blood flow. Detailed data collection and treatment are provided in the SI (Section S2). We derived interspecific (between-species) allometries for relationships between volumes of fish tissue *i* (*V_i_*, mL) and body mass (*M*, g):
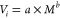
1where *a* is the allometric coefficient and *b* is the allometric exponent (Table S2, SI). For each tissue, the values of *a* and *b* were determined by fitting a straight line to log-transformed values for *V_i_* and *M* using linear regressions (Figure S1, SI). The difference between the total and summed volumes of other compartments was assumed to be muscle.

Lipid and water contents in respective fish tissues (expressed as a percentage of tissue volumes) were obtained from the literature for 22 fish species (Section S2). Average compositions are summarized in Table 1. The remaining contents were assumed to be non-lipid organic matter (NLOM; i.e., proteins and carbohydrates).

**Table 1:** Summary of Tissue Content (% of Tissue Volume, Geometric Mean, and ± 1 Standard Deviation) and Weighting Factors in Fish

tissue	neutral lipid content (%)	polar lipid content (%)	water content (%)	non-lipid organic matter content (%)	weighting factor (see eq 3)
adipose fat	88.4 (±7.2)	0.9 (±0.1)	5.9 (±3.7)	4.8	0.626
blood	0.8 (±0.2)	0.7 (±0.2)	85.1 (±1.5)	13.4	-
brain	4.7 (±0.7)	4.3 (±0.6)	68.2 (±5.7)	22.8	3.600
gastrointestinal tract	5.2 (±1.4)	2.3 (±0.6)	67.0 (±10.3)	25.5	4.846
gonads	4.2 (±2.7)	2.4 (±1.5)	67.2 (±4.8)	26.2	3.367
kidney	5.9 (±3.8)	2.3 (±1.5)	70.3 (±10.9)	21.5	7.005
liver	3.7 (±1.6)	2.5 (±1.1)	70.4 (±5.7)	23.4	2.230
poorly perfused tissues	2.4 (±6.3)	0.9 (±2.4)	76.9 (±3.8)	19.9	0.733
richly perfused tissues	6.6 (±3.7)	0.8 (±0.5)	58.7 (±10.3)	33.8	3.600
skin	3.7 (±1.3)	1.2 (±0.4)	69.2 (±5.1)	25.9	0.570

The cardiac output (*F*_card_, mL/day) and oxygen consumption rate (*VO*_2_, mg O_2_/day), scaling with the 3/4-power of body mass and increasing exponentially with temperature within the biologically relevant range (0–40 °C) were shown to be well approximated by the Boltzmann–Arrhenius equation:^17,18,32^
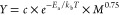
2where *Y* represents the variables *F*_card_ and *VO_2_*, *c* is the constant (mL/day/g or mg O_2_/day/g), *E*_a_ is the average activation energy for rate-limiting enzyme-catalyzed biochemical reactions (J), *k*_b_ is the Boltzmann constant (J/K), *T* is the absolute temperature (K), *M* is the body mass (g), and 0.75 is the allometric exponent.^17,33,34^ Mass-normalized *F*_card_ and *VO*_2_ (i.e., ln(*Y*/*M*^0.75^)) were plotted against inverse *T* (1/*T*) to determine the respective slopes (−) and intercepts (ln *c*) of regressions (Table S3, SI).

Empirical data on blood distribution over different tissues in fish are limited. Therefore, we assumed that the distribution of cardiac output to each tissue is proportional to the tissue volume, accounting for differences among tissues by a weighting factor (*WF*, summarized in Table 1).^35,36^*WF*s were based on measurements in rainbow trout (Section S2) as a reference for other fish species (e.g., refs (36)(37), and (35)). To ensure that the sum of blood flows to tissues equals *F*_card_, the relative blood flow was normalized (Table S1).

#### 2.2.2 Biochemical Parameters

Biochemical parameters include chemical absorption and elimination rate constants in respective compartments, partitioning properties, and hepatic clearance.

We followed the classical fugacity theory to estimate the exchange coefficient for chemical flux at fish gills (*k_x_*, mL/day). Regarding the ionization potential of pharmaceuticals, the ionized fraction typically exhibits lower permeability through membranes than the neutral fraction,^38^ leading to lower lipophilicity. Therefore, we replaced *K*_OW_ with the octanol–water distribution coefficient (*D*_OW_), combining the neutral (*K*_OW_) and the ionic molecules’ (*K*_OW,ion_) contributions. *k_x_* was shown to be allometrically correlated to fish body mass (*M*, g) and regulated by resistance in water (ρ_H2O_ = 2.8 × 10^–3^ day·kg^0.75^/L) and lipid layers (ρ_CH2_ = 68 day·kg^0.75^/L) and gill ventilation (γ_water_, L/kg^0.75^/day).^20^ We added the parameter of blood perfusion (γ_blood_, L/kg^0.75^/day) to reflect the blood flow delay as shown in^39^
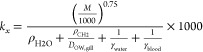
3where γ_water_ is the mass-normalized oxygen consumption rate combined with dissolved oxygen concentration^39^ and γ_blood_ is the mass-normalized cardiac output combined with the blood:water partition coefficient (Table S1). The pH at the gill surface was assumed to be the same as that in water, and the calculation of *D*_OW,gill_ is shown in Table S1. The assimilation rate constant from food (*k*_u_, day^–1^), assimilated fraction of food (*f*_abs_, −), and elimination rate constant of feces (*k*_e,feces_, day^–1^) were analogously derived (Table S1). Compared to water expiration from gills, excretion from urine was assumed to be negligible. The unbound fraction in blood (*UF*, −) was calculated from the plasma:water distribution ratio (*D*_plasma:w_, correlated with percent plasma protein binding and estimated with regression^23^):
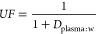
4

Tissue *i*:water partition coefficients (*P*_*i*:W_) were the sum of substance distributions over different fish components (i.e., lipids, NLOM, and water) and water:^21,40^
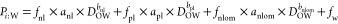
5where *D*_OW_ was calculated at biological pH (i.e., 7.4 in the whole fish body); *f*_nl_, *f*_pl_, *f*_nlom_, and *f*_w_ are the fractions of the neutral lipid, polar lipid, NLOM, and water in respective tissues, respectively (Table 1); and parameters *a* and *b* were regression constants and slopes for each tissue component obtained from a previous study.^21^ Tissue *i*:blood partition coefficients (*P*_*i*:B_) were calculated as the ratio between the tissue *i*:water partition coefficient (*P*_*i*:W_) and the blood:water partition coefficient (*P*_B:W_).

A QSAR model was developed by Arnot and co-workers^22^ to provide size- and temperature-normalized estimates of whole-body primary biotransformation half-lives (*HL*_N_, day) in fish, included in EPI Suite v4.11.^41^ The liver was assumed to be the main organ responsible for biotransformation in fish.^42^ We obtained *HL*_N_ values from EPI Suite (Table S4, SI) and calculated hepatic clearance (*Cl*_hepatic_, mL blood/day/g fish):^22,43^
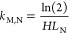
6a
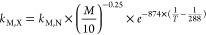
6b
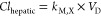
6cwhere *k*_M,N_ and *HL*_N_ are the biotransformation rate constant (day^–1^) and half-life (day) normalized to a 10 g fish at 15 °C (288 K), respectively, *k*_M,X_ is the study-specific rate constant (day^–1^) corrected for body mass and temperature differences, *M* is the study-specific fish body mass (g), and *T* is the study-specific water temperature (K), and *Cl*_hepatic_ is the hepatic clearance (mL blood/day/g fish). *V*_D_ (mL blood/g fish) is considered as the capacity of fish to accumulate chemical relative to that of blood, estimated following the approach outlined by Nichols et al.^44^ (Table S1). As the QSAR^22^ included few substances with appreciable ionization at physiological pH, we also applied measured biotransformation HL values^45−47^ after correcting for body mass and temperature differences (eq 6a–c).

### 2.3 Evaluation Data

Model performance was evaluated with measured concentrations of pharmaceuticals in respective tissues in fish. Details on the literature search and filtering procedures are described in the SI (Section S3). In total, 10 publications covering 6 fish species and 273 measurements were included in our dataset (Table S5, SI), covering only respiratory uptake routes (aqueous exposure). We also estimated steady-state BCFs using our model and compared these values with observed steady-state BCFs in cyprinoid fish^5^ and zebrafish^45^ to evaluate the predictive ability of the model.

### 2.4 Model Evaluation

Fold difference (FD) was calculated to assess model performance:
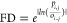
7where *p_i,j_* and *o_i,j_* are the predicted and observed concentrations of chemical *i* in organ *j*, respectively.

## 3 Results

### 3.1 Parametrization

**Figure 2:**
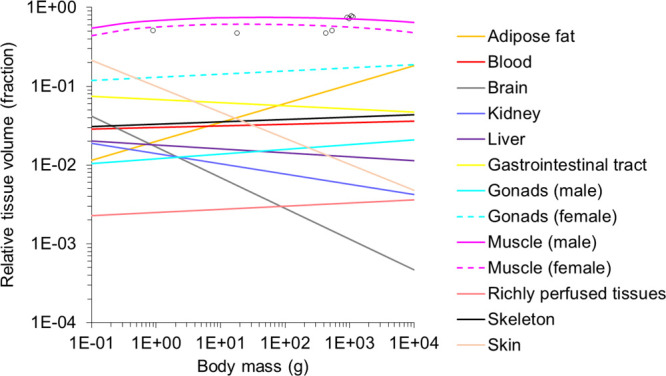
Relative tissue volume (fraction of whole-body mass) versus body mass (g). Muscle volume was assumed to be the difference between the total volume and the summed volumes of other compartments. Open circles are the measured relative muscle volume.^37,48−50^ Derived regressions are listed in Table S2.

Tissue volume was scaled allometrically to body mass (*V* = *aM^b^*) with slopes (*b*) between 0.61 and 1.24 (Table S2). Fractions of support and transport organs in whole fish (blood, GIT, gonads, muscle, skeleton, and RPT) are size-independent (nearly horizontal lines in Figure 2, *b* ≈ 1). The relative fraction of the brain, skin, kidney, and liver declined with the increasing body mass (*b* < 1), where the relative fraction of the brain exhibited the greatest decrease (*b* = 0.61; 95% CI: 0.58–0.64). The relative fraction of adipose fat increased with the body mass with *b* = 1.24 (1.13–1.36).

**Figure 3:**
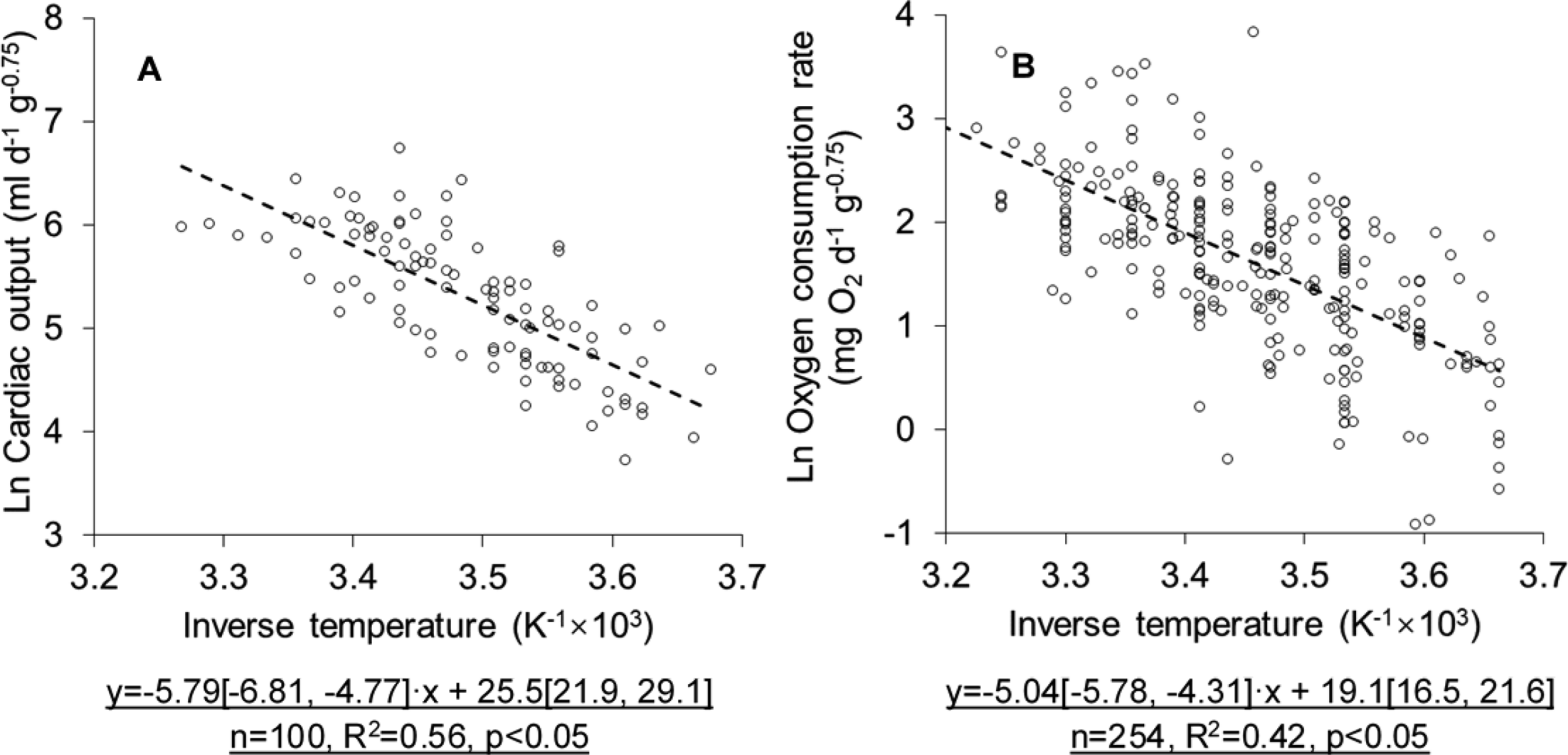
Mass-normalized (A) cardiac output and (B) oxygen consumption rate versus inverse temperature (1/*T*, K^–1^) based on the Boltzmann–Arrhenius equation.

The mass-normalized cardiac output and oxygen consumption rate were estimated as a function of temperature (Figure 3). Collected data were described by straight lines with similar slopes, indicating similar activation energies required for cardiac output and oxygen consumption. There appeared a clear gap in mass-normalized cardiac output data below the regression line (Figure 3A); see discussion in Section 4.1.1. The derived slopes agreed well with previous studies (−5.02^17,32^).

### 3.2 Comparison with Measured Concentrations

**Figure 4:**
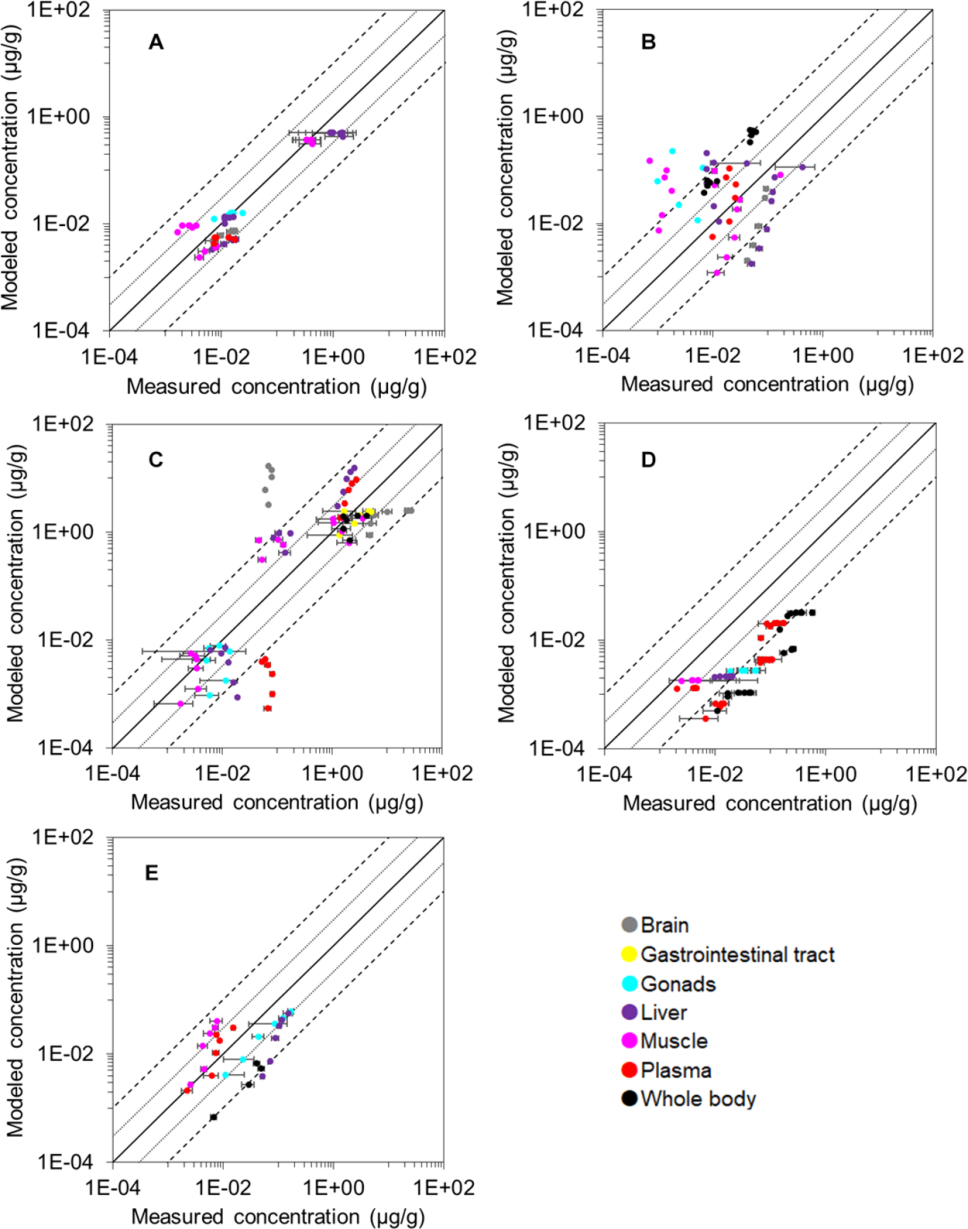
Comparison of modeled concentrations (μg/g) with measured concentrations (μg/g) for (A) carbamazepine, (B) diclofenac, (C) ibuprofen, (D) diphenhydramine, and (E) fluoxetine. Modeled concentrations were based on the biotransformation half-life derived from EPI Suite v4.11.^22^ Organs are classified by colors. Means and standard errors are shown. Dotted and dashed lines represent the 3-fold and 10-fold differences, respectively.

Two sets of results (I and II) were computed. Set I was estimated based on biotransformation HLs from EPI Suite and set II based on measured HLs. Results of set I are shown in Figure 4 (time-series comparisons shown in Figures S2–S6, SI). Overall, 73 and 41% of the estimations were within 10- and 3-FD from measurements, respectively (Table S6, SI). Model accuracy varied from one compound to another. For the neutral carbamazepine, acidic ibuprofen, and basic fluoxetine, the model provided reasonable estimations with at least 80 and 50% of predictions within 10- and 3-FD, respectively (Table S6). For the acidic diclofenac, 64 and 24% of the estimations were within 10- and 3-FD, respectively. The model systematically underestimated the basic diphenhydramine concentrations in fish (Figure 4D).

**Figure 5:**
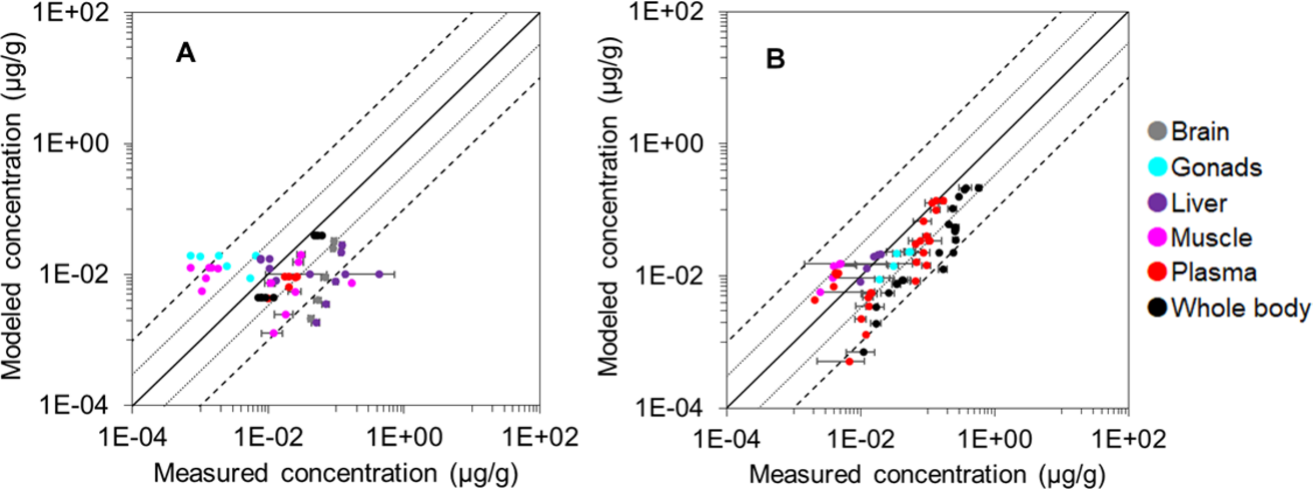
Comparison of modeled concentrations (μg/g) with measured concentrations (μg/g) for (A) diclofenac and (B) diphenhydramine. Modeled concentrations were based on the measured biotransformation half-life from the literature. Organs are classified by colors. Means and standard errors are shown. Dotted and dashed lines represent the 3-fold and 10-fold differences, respectively.

When experimental biotransformation HLs were applied, 87 and 59% of the estimations were within 10- and 3-FD from measurements, respectively (full results in Figures S7–S12, SI). The results improved for diclofenac and diphenhydramine (Figure 5): 51 and 57% of the estimations were within 3-FD for diclofenac and diphenhydramine, respectively. No apparent changes were observed for other pharmaceuticals due to similar estimated and measured biotransformation HLs after correcting for body mass and temperature differences. For both sets of results at the tissue level, the estimated brain concentrations were the least accurate: on average, 40% of estimations deviated from measurements by more than a 10-fold (Table S6).

Steady-state BCFs were calculated (Figure S13, SI). Here, 70 and 88% of the estimations were within 10-FD from measurements based on biotransformation HLs from EPI Suite and the literature, respectively.

## 4 Discussion

We developed a generalized fish PBK model and evaluated its performance for pharmaceuticals. Our generalized model focused on parameterization as functions of biological traits and chemical properties and explicitly accounted for ionization. Below, we discuss key elements of our study, i.e., parameterization, prediction capacity, and potential model use.

### 4.1 Parameterization

#### 4.1.1 Physiological Parameters

Allometric scaling, describing the effects of body size on biological processes, is a fundamental principle widely used in biology.^33^ We correlated tissue volumes with fish body mass (*V* = *aM^b^*, *p* < 0.05). The allometric exponents *b* of all tissues except for adipose fat are ≤1, suggesting that the relative volume of most organs remained the same or declined with the increasing body size. The relative brain volume exhibited the greatest decrease (*b* = 0.61). The results were consistent with previous studies for specific species.^51−54^ Cardiac output and oxygen consumption were correlated with fish body mass through allometric scaling, and the Boltzmann–Arrhenius equation was applied to adjust for temperature differences (Figure 3, *p* < 0.05). It should be noted that cardiac output data cover fish sizes from 89 g to 3.75 kg. Data above the regression (Figure 3A) generally represent larger fish than those below, supporting the tenet that larger fish tend to have higher cardiac outputs than smaller ones. The apparent gap in data below the regression line may suggest the effect of allometry on cardiac function between fish smaller than 600 g and those larger. Additionally, mass-normalized oxygen consumption data span several natural logarithm units around the regression line (Figure 3B), which was shown to be explained by taxonomic variation not captured by our interspecific relationships.^32^

Our generalized PBK model aims to allow extrapolating physiological parameters based on size and temperature differences, thus requiring an overall interspecific rather than intraspecific scaling. However, given the large variation around the regressions as shown in Figure 3, a global sensitivity analysis using probabilistic approaches is highly recommended to assess the impact of this variability and uncertainty on PBK predictions. Such analysis may support the identification of fish taxa with higher internal exposure and thus risk.

With a similar aim to expand the applicability domain of PBK models, a recent work^14^ employed a probabilistic approach (Monte Carlo-like simulations) to predict required physiological parameters, using statistical distributions derived from 69 freshwater fishes in Canada. On the one hand, while such distributions adequately capture inter- and intraspecies variabilities of individual model parameters, it may result in combinations of parameter values that are biologically implausible, leading to non-representative model predictions hindering mechanistic interpretation. On the other hand, while our parameterization based on a few key descriptors and overarching principles improves understanding and allows extrapolation across a broad range of conditions, substances, and species, it does not capture all inter- and intraspecies variabilities since the variability of some parameters is ignored. For instance, body mass and temperature could explain approximately 50% of the variance in cardiac outputs and oxygen consumption rates (Figure 3). Obviously, lumping variance caused by the body mass and temperature in a single statistical distribution will lead to less accurate estimations. Yet, variabilities not explained by the descriptors can be expressed as residuals allowing stochastic simulations (e.g., Monte Carlo). In other words, mechanistic and probabilistic approaches could supplement each other, improving the accuracy of model parameterization.

#### 4.1.2 Biochemical Parameters

The accumulation potential of a compound depends on both species and chemical attributes, as illustrated in the parametrization of absorption (i.e., exchange coefficient) and sorption capacity (i.e., partition coefficients). Due to limited experimental data on uptake and distribution of ionizable substances (pharmaceuticals) in fish, calibrating existing regressions is difficult. Nevertheless, estimated exchange coefficients of carbamazepine, diclofenac, diphenhydramine, and fluoxetine calculated in our study for a 1 g zebrafish at 25 °C (22.5, 6.8, 12.0, and 5.6 mL/day, respectively) were comparable to the measured values (22.2, 14.2, 5.3, and 2.2 mL/day, respectively).^45^ An exception was ibuprofen as our estimated exchange coefficient for zebrafish (1.0 mL/day) was more than 350 times lower than the measurement (357 mL/day). This deviation is also reflected in the large deviation between estimated and measured concentrations of ibuprofen in fish plasma (Figures S4A and S10A). The blood:water partition coefficient (*P*_B:W_) estimated based on *D*_OW_ in the present study agreed well with the empirical equation developed for rainbow trout^55^ (Figure S14, SI). The estimated *P*_B:W_ of diphenhydramine in fathead minnow (8.9 at biological pH 7.4) was in the range of measurements (1.4–16.7).^26^ Additionally, predicted tissue-blood partition coefficients of carbamazepine, diclofenac, and diphenhydramine in cyprinoids were generally in reasonable agreement with the measured values^5^ (Table S7, SI).

Several uncertainties should be considered regarding biochemical parameter estimations. While neutral chemicals primarily partition to neutral (storage) lipids, ionizable substances sorb more strongly to polar lipids and proteins.^56,57^ We roughly took this factor into account by utilizing generalized terms of 0.3·*D*_OW_, 2.0·*D*_OW_^0.94^, and 2.9·*D*_OW_^0.63^ for distribution of ionizable substances over neutral lipids, polar lipids, and proteins, respectively.^21^ The terms correspond to a factor of 6.7 greater sorption to polar lipids compared to neutral lipids when log *D*_OW_ = 0 and a factor of 5 greater sorption to polar lipids than to neutral lipids when log *D*_OW_ = 2. This sorption difference is somewhat smaller than observed lipophilicity profiles of drugs in octanol–water and membrane–water systems,^58^ especially for chemicals predominantly charged at physiological pH (e.g., acids with p*K*_a_ < 5 and bases with p*K*_a_ > 9). Previous studies^45,57^ proposed using the membrane–water distribution coefficient (*D*_MW_), explicitly considering the distribution of ionized form to polar lipids. When applying available *D*_MW_ values for diclofenac and ibuprofen,^58^ we only observed marginal differences in bioaccumulation as the difference of tissue:blood partitioning was less than 2.2 times for all tissues including the whole body except for adipose tissue (5 times difference, details in Section S4, SI). Due to data limitations, more specific partitioning parameters (e.g., *D*_MW_) were not incorporated in the current model, which might provide better estimates of chemical partitioning. Given the potential deviations in partitioning behavior of ionizable substances between estimates and measurements, it is recommended to understand the partitioning mechanisms before applying other proxies for predicting ionic interactions.

Variabilities of both the gill surface and biological pH were not considered in the model. In the present study, it was assumed that the pH of the absorption site (gill surface) was the same as water pH, and the pH inside fish remained constant at 7.4. However, pH at the gill surface can be reduced due to the elimination of metabolically produced acids (e.g., conversion of excreted CO_2_ to HCO_3_^–^ and H^+^) secreted to respired water and substantially reducing the pH of inspired water.^59−61^ pH depression at the gill surface is especially relevant for acidic chemicals as absorption rates and toxicity would increase at low pH due to an increase in the proportion of neutral form of the chemical. Our model clearly illustrates this “pH depression effect”. When the pH at the gill interface was set one pH unit lower than the water pH (the same conditions^45^), uptake and concentrations of ibuprofen for zebrafish in respective tissues were 5.6 and 5.3 times higher, respectively. Additionally, pH in fish compartments may also fluctuate. One example is the post-prandial “alkaline tide” (elevated pH and HCO_3_^–^ in blood due to gastric acid secretion following a meal), well-characterized in air-breathing animals (mammals and reptiles).^62^ However, it has limited and contrary evidence in fish.^63,64^ Nevertheless, as pH differences among fish compartments may lead to different sorption capacities of pharmaceuticals and internal concentrations, it is important to include this factor in future analysis.

Biotransformation HLs and corresponding biotransformation rate constants are considered key parameters in determining the bioaccumulation potential of hydrophobic chemicals in fish.^43,65,66^ To date, *in silico* tools (e.g., QSARs) have been developed to predict *in vivo* biotransformation in fish.^22,67,68^ The developed biotransformation QSARs provide insight into the potential role of metabolism. However, due to a lack of measured biotransformation data on ionizable substances, QSARs were mainly developed for neutral ones. Consequently, biotransformation estimations in our study for ionizable substances (even after correcting for size and temperature differences) are subject to high uncertainties. For example, *in vitro* studies indicated that diphenhydramine displayed no significant depletion by rainbow trout S9 fractions,^26,69^ while the HL derived from EPI Suite^22^ for diphenhydramine was short (0.05 day). Our study also showed better model performance when applying experimental biotransformation HLs rather than QSAR-estimated values for diphenhydramine, with a longer time to reach higher steady-state concentrations in tissues and the whole body (Figures S5 and S11). Consequently, more empirical data on biotransformation of ionized chemicals in fish are needed from *in vitro* experiments to refine the current QSAR models and improve biotransformation estimations.

Given the complexity addressed in the present study, we did not perform sensitivity analysis to identify the most influential parameters on the model’s outputs. Alternatively, we summarized the estimated exchange coefficient (mL/g/day) and hepatic clearance (mL/g/day) for each evaluation study (Figure S15, SI), regarded as important bioaccumulation kinetics. The data showed positive relationships between exchange coefficients and *D*_OW_ values: when *D*_OW_ increased two orders of magnitude, the uptake also increased to a similar extent. Biotransformation HLs explained the main variance in hepatic clearance with negative relationships (Figure S15). Consequently, with similar concentrations in the ambient environment, chemicals with higher *D*_OW_ and biotransformation HL values are expected to have higher concentrations in tissues and take longer to reach a steady state. Additionally, as shown in Section S4, tissue:blood partitioning explained the concentration differences among tissues (higher concentrations are expected in tissues with higher tissue:blood partitioning).

### 4.2 Prediction Capacity of the Model

With biotransformation HLs from EPI Suite, 73, 54, and 41% of the predicted concentrations were within a 10-, 5-, and 3-FD from measurements, respectively. With experimental biotransformation HLs, the performance improved to 87, 73, and 59%, respectively. For species-specific PBK models developed by others,^29,35,50^ 75–90% of estimations were typically within 5-FD from measurements (Table S8, SI). It should be noted that the performance of these species-specific PBK models was evaluated primarily for neutral chemicals, while large deviations were reported for ionizable substances. In our study, 76% of estimations were within 10-FD for ionizable substances. By contrast, Stadnicka and co-workers^29^ reported that all predicted internal concentrations for ionizable compounds (phenol, 2,4,5-trichlorophenol, 4-nitrophenol, and C12LAS) deviated by more than a 10-fold. Brinkman and co-workers^50^ indicated that most PBK models to date do not yield accurate predictions for ionizable compounds. Additionally, the prediction accuracy of our generalized model was similar to the model applied to four fish species^30^ (83 and 57% of estimations within 10- and 3-FD from measurements, respectively), while Grech et al.’s model^30^ largely relied on measured physiological parameters for specific species. Therefore, our model not only applies to many more species but is also more accurate for ionizable compounds than any of the existing species-specific PBK models.

Predicted brain concentrations were less accurate than other organs since 40% of the predictions deviated from measurements by more than a 10-fold. Such discrepancies are also common in PBK studies by others (e.g., refs (30) and (70)). For instance, 55% of the predicted brain concentrations deviated by more than a 10-fold for ionizable substances (bisphenol A and oxytetracycline) in Grech et al.’s model.^30^ The large deviation of brain concentrations may be explained by the role of plasma protein binding in the transport across the blood–brain barrier and the brain uptake of the drug: plasma protein binding limits brain uptake by reducing the free fraction of substances in the circulation.^71,72^ Such selective transport can only be considered when partition coefficients have been measured since they are poorly described by QSAR models.^30^

Next to a direct comparison of time-course concentrations in tissues, 88% of the estimated steady-state BCFs by our model were within 10-FD from measurements based on measured biotransformation HLs (Figure S13B). Notably, this accuracy is similar to the aforementioned multispecies PBK model using a probabilistic approach,^14^ with 82% of estimated BCFs within 10-FD for natural organic chemicals. Our model further illustrated the pH effects on the accumulation of ionizable substances in fish. When fathead minnows were exposed to diphenhydramine at pH 6.7, 7.7, and 8.7 (same conditions^26^), estimated steady-state BCFs in the whole body were 1.0, 5.6, and 24.4 L/kg, respectively. When Japanese medakas were exposed to fluoxetine at pH 7, 8, and 9 (same conditions^27^), estimated steady-state BCFs were 3.6, 10.7, and 46.1 L/kg, respectively. These BCF estimations were comparable to measurements (all within 5-FD),^26,27^ although there was a tendency to underestimate measured values. The discrepancy could be explained by the variation in taxonomy, partitioning behavior of ionizable substances, and biotransformation rates that have already been discussed above. Previous studies^57,73^ also compiled the whole-body BCFs for ionizable organic chemicals across various properties. However, given the feature of PBK models providing time-course concentrations in specific tissues other than the whole body and the uncertainties listed above, an extensive comparison of estimated and measured BCFs is beyond the scope of this study.

### 4.3 Potentials of the Model in Environmental Risk Assessment

Our generalized fish PBK model focuses on parametrization in a mechanism-based approach and provides reasonable internal concentrations, especially for ionizable substances. By estimating input parameters mechanistically, the PBK modeling process becomes (far) less intensive in terms of data requirements. Additionally, our *in silico* approach supports less animal testing and the “Replacement, Reduction, and Refinement” principles related to animal welfare. Moreover, the flexible model structure facilitates its application in chemical risk assessments for different fish, chemicals, and aquatic environment conditions (pH and temperature) of concern. Given countless substances and species, the development of generalized PBK models is inevitable and favorable in terms of efficiency and feasibility.

Within regulatory contexts, the BCF is an important bioaccumulation indicator required by the Registration, Evaluation, Authorisation, and Restriction of Chemical substances regulation^74^ in Europe. Both one- (assuming whole organism as a single well-mixed compartment) and multicompartment (e.g., the PBK model) can predict whole-body chemical concentrations and thus BCFs. Previous studies^29,43,75,76^ have indicated that the accuracy of simple one-compartment models appeared to be comparable or only marginally lower compared to that of complex PBK models. However, PBK models are essential to estimate time-course chemical concentrations in potentially targeted tissues, especially relevant to pharmaceuticals acting on specific tissues. With this information, safe chemical intake levels and adverse endpoints can be derived. PBK models can also help in facilitating quantitative *in vitro* to *in vivo* extrapolation approaches.^77^ Moreover, PBK models are indispensable when non-instantaneous processes dominate (e.g., delayed enzyme or transporter induction).

It should be emphasized that the model was evaluated for six fish species and five pharmaceuticals via aqueous uptake based on the available evaluation data. The evaluation should be expanded to more species, substances, and exposure routes as more data become available. Additionally, a global sensitivity analysis should also be performed to determine the most influential input parameters and the impacts of variabilities on the model output. Furthermore, more empirical data on ionizable substances’ biotransformation and partitioning in fish are needed to refine the current QSAR models and improve estimations. We encourage further refinement and application of our generalized fish PBK model in chemical risk assessment for fish, given the ultimate goal of protecting the whole ecosystem in the context of animal health, ecosystem integrity, and food safety.

## Supporting Information Available

(Section S1) PBK model description; (Section S2) physiological data collection and treatment; (Section S3) evaluation data search and filtering procedures; (Section S4) comparison of internal concentrations of diclofenac and ibuprofen using two partitioning estimation approaches; (Figure S1) fish organ/tissue mass relative to fish body mass; (Figures S2–S6) time series of measured carbamazepine, diclofenac, ibuprofen, diphenhydramine, and fluoxetine concentrations in fish organs and estimated concentrations based on biotransformation half-life from EPI Suite, respectively; (Figure S7) comparison of modeled concentrations with measured concentrations for all pharmaceuticals based on the measured biotransformation half-life from the literature; (Figures S8–S12) time series of measured carbamazepine, diclofenac, ibuprofen, diphenhydramine, and fluoxetine concentrations in fish organs and estimated concentrations based on biotransformation half-life from the literature, respectively; (Figure S13) comparison of modeled bioconcentration factors (BCFs) with measured BCFs from the literature; (Figure S14) comparison of the estimated blood:water partition coefficient from the present study and Fitzsimmons et al.; (Figure S15) correlation of bioaccumulation kinetics and fish and chemical properties; (Table S1) summary of model inputs and parameters used in the present fish PBK model; (Table S2) regression summary of organ/tissue volumes related to fish body mass; (Table S3) regression summary of temperature-dependent parameters related to fish body mass and/or absolute temperature; (Table S4) physicochemical properties of selected pharmaceuticals; (Table S5) validation data; (Table S6) % of predictions within 10- and 3-fold differences (number of measurements); (Table S7) comparison of estimated and measured tissue-blood partition coefficients; and (Table S8) summary of existing fish PBK models (PDF)
