# Supplementary material for: A Generalized Physiologically Based Kinetic Model for Fish for Environmental Risk Assessment of Pharmaceuticals
Source: Environ Sci Technol. 2022 Apr 26;56(10):6500–10. doi: 10.1021/acs.est.1c08068 (PMC9118555; doi:10.1021/acs.est.1c08068)
Supplement: Supplementary file 1 — es1c08068_si_001.pdf [file es1c08068_si_001.pdf]

Supporting Information

for 'A generalised physiologically based kinetic model for fish for environmental risk assessment of pharmaceuticals'

Jiaqi Wang[1*], Tom M. Nolte[1], Stewart F. Owen[2], Rémy Beaudouin[3], A. Jan Hendriks[1], Ad M.J. Ragas[1,4]

[1] Department of Environmental Science, Radboud Institute for Biological and Environmental Sciences, Radboud University, Nijmegen, 6500 GL, the Netherlands

[2] AstraZeneca, Global Sustainability, Macclesfield, Cheshire, SK10 2NA, United Kingdom

[3] Institut national de l'environnement industriel et des risques (INERIS), Verneuil-en-Halatte, 60550, France

[4] Department of Environmental Sciences, Faculty of Science, Open University, Heerlen, 6419 AT, the Netherlands

* Corresponding author. E-mail address: jiaqi.wang@ru.nl

Number of pages: 44

Number of figures: 15

Number of tables: 8

Section S1 PBK model

The model consists of eleven compartments: arterial and venous blood, gastrointestinal tract (GIT), skin, kidney, fat, liver, gonads, brain, poorly perfused tissues (PPT; skeleton and muscles) and richly perfused tissues (RPT; the remaining viscera including heart, spleen, etc.). All compartments were assumed well-mixed with a blood flow-limited distribution. The model assumes that absorption of a pharmaceutical takes place either via the gills (in case of water inspiration) or the GIT (in case of food ingestion). Venous blood out of RPT and gonads was assumed to join the portal vein and enter the liver [1]. A fraction of venous blood out of the skin and PPT (90% and 60%, respectively) was assumed to enter directly into the kidney, with the remaining contributing to the mixed venous circulation [2]. Elimination can occur via urine, faeces, expiration and hepatic metabolism.

Table S1 Summary of model inputs and parameters used in the present fish PBK model[a]

Symbol	Unit	Description	Value/Equation	Reference
M	g (ml)	Fish body mass (volume)	Model input, Table S5	-
log K_{OW}	-	Octanol-water partitioning coefficient of the neutral molecule	Model input, Table S4	-
log $K_{OW,ion}$	-	Octanol-water partitioning coefficient of the ionic molecule	Model input, Table S4	-
pKa	-	Acid dissociation constant	Model input, Table S4	-
HL_N	d	Whole-body primary biotransformation half-life normalised to a 10-g fish at 15 ℃ (288 K)	Model input, Table S4	-
T	K	Water temperature	Model input, Table S5	-
pH_w	-	pH in water/at gill surface	Model input, Table S5	-
C_{water}	µg/ml	Dissolved chemical concentration in inspired water	Model input, Table S5	-
Q_{ingest}	µg/d	Chemical quantity administered in gastrointestinal tract	Model input	-
α_{Fpp}	-	Fraction of PPT blood flow to venous blood	0.4	[2]

α_{Fs}	-	Fraction of skin blood flow to venous blood	0.1	[2]
$f_{n,gill}$	-	Fraction of the neutral molecule at gill surface	$f_{n,gill} = \frac{1}{1 + 10^{i \cdot (pH_w - pK_a)}}$ (i = 1 for acid and -1 for base)	[3]
$f_{n,fish}$	-	Fraction of the neutral molecule in fish body	$f_{n,fish} = \frac{1}{1 + 10^{i \cdot (7.4 - pK_a)}}$ (i = 1 for acid and -1 for base)	[3]
$D_{OW,gill}$	-	Octanol-water distribution coefficient at gill surface pH	$D_{OW,gill} = f_{n,gill} \cdot K_{OW} + (1 - f_{n,gill}) \cdot K_{OW,ion}$	[4]
D_{OW}		Octanol-water distribution coefficient at fish body pH 7.4	$D_{OW} = f_{n,fish} \cdot K_{OW} + (1 - f_{n,fish}) \cdot K_{OW,ion}$	[4]
V_i	g (ml)	Mass (volume) of organ i	Table S2	Present study
F_{card}	ml/d	Cardiac output	$F_{card} = e^{25.5} \cdot e^{\frac{-5790}{T}} \cdot M^{0.75}$	Present study
WF_i	-	Weighting factor of compartment *i*	Table 1	Present study
F_i	ml/d	Blood flow to compartment *i*	$F_i = \frac{WF_i \cdot \frac{V_i}{M}}{\sum_{i=1}^{9}(WF_i \cdot \frac{V_i}{M})} \cdot F_{card}$	Present study
VO_2	mg O_2/d	Oxygen consumption rate	$VO_2 = e^{19.1} \cdot e^{\frac{-5040}{T}} \cdot M^{0.75}$	Present study
Cox	mg O_2/ml	Dissolved oxygen concentration	$C_{ox} = e^{-10.6} \cdot e^{\frac{1743.3}{T}}$	Present study

$f_{nl,i}$	-	Fraction of neutral lipid in compartment *i*	Table 1	Present study
$f_{pl,i}$	-	Fraction of polar lipid in compartment *i*	Table 1	Present study
$f_{nlom,i}$		Fraction of NLOM in compartment *i*	Table 1	Present study
$f_{w,i}$		Fraction of water in compartment *i*	Table 1	Present study
$P_{B:W}{}^{b}$	-	Blood: water partition coefficient	$P^{B:W} = 0.008 \cdot K_{OW} + 0.007 \cdot K_{OW}{}^{0.94} + 0.134 \cdot K_{OW}{}^{0.63} + 0.851$ (Neutral substances)<br>$P^{B:W} = 0.008 \cdot 0.3 \cdot D_{OW} + 0.007 \cdot 2.0 \cdot D_{OW}{}^{0.94} + 0.134 \cdot 2.9 \cdot D_{OW}{}^{0.63} + 0.851$ (Ionic substances)	[5]
$P_{go:B}{}^{b}$	-	Gonad: blood partition coefficient	$P^{go:B} = \frac{0.042 \cdot K_{OW} + 0.024 \cdot K_{OW}{}^{0.94} + 0.262 \cdot K_{OW}{}^{0.63} + 0.672}{P^{B:W}}$ (Neutral substances)<br>$P^{go:B} = \frac{0.042 \cdot 0.3 \cdot D_{OW} + 0.024 \cdot 2.0 \cdot D_{OW}{}^{0.94} + 0.262 \cdot 2.9 \cdot D_{OW}{}^{0.63} + 0.672}{P^{B:W}}$ (Ionic substances)	[5]
$P_{k:B}{}^{b}$	-	Kidney: blood partition coefficient	$P^{k:B} = \frac{0.059 \cdot K_{OW} + 0.023 \cdot K_{OW}{}^{0.94} + 0.215 \cdot K_{OW}{}^{0.63} + 0.703}{P^{B:W}}$ (Neutral substances)<br>$P^{k:B} = \frac{0.059 \cdot 0.3 \cdot D_{OW} + 0.023 \cdot 2.0 \cdot D_{OW}{}^{0.94} + 0.215 \cdot 2.9 \cdot D_{OW}{}^{0.63} + 0.703}{P^{B:W}}$ (Ionic substances)	[5]
$P_{l:B}{}^{b}$	-	Liver: blood partition coefficient	$P^{l:B} = \frac{0.037 \cdot K_{OW} + 0.025 \cdot K_{OW}{}^{0.94} + 0.234 \cdot K_{OW}{}^{0.63} + 0.704}{P^{B:W}}$	[5]

			$P^{l:B} = \frac{0.037 \cdot 0.3 \cdot D_{OW} + 0.025 \cdot 2.0 \cdot D_{OW}^{0.94} + 0.234 \cdot 2.9 \cdot D_{OW}^{0.63} + 0.704}{P^{B:W}}$ (Neutral substances) (Ionic substances)	
$P_{pp:B}{}^{b}$	-	Poorly perfused tissue: blood partition coefficient	$P^{pp:B} = \frac{0.024 \cdot K_{OW} + 0.009 \cdot K_{OW}^{0.94} + 0.199 \cdot K_{OW}^{0.63} + 0.769}{P^{B:W}}$ (Neutral substances) $P^{pp:B} = \frac{0.024 \cdot 0.3 \cdot D_{OW} + 0.009 \cdot 2.0 \cdot D_{OW}^{0.94} + 0.199 \cdot 2.9 \cdot D_{OW}^{0.63} + 0.769}{P^{B:W}}$ (Ionic substances)	[5]
$P_{rp:B}{}^{b}$	-	Richly perfused tissue: blood partition coefficient	$P^{rp:B} = \frac{0.066 \cdot K_{OW} + 0.008 \cdot K_{OW}^{0.94} + 0.338 \cdot K_{OW}^{0.63} + 0.587}{P^{B:W}}$ (Neutral substances) $P^{rp:B} = \frac{0.066 \cdot 0.3 \cdot D_{OW} + 0.008 \cdot 2.0 \cdot D_{OW}^{0.94} + 0.338 \cdot 2.9 \cdot D_{OW}^{0.63} + 0.587}{P^{B:W}}$ (Ionic substances)	[5]
$P_{s:B}{}^{b}$	-	Skin: blood partition coefficient	$P^{s:B} = \frac{0.037 \cdot K_{OW} + 0.012 \cdot K_{OW}^{0.94} + 0.259 \cdot K_{OW}^{0.63} + 0.692}{P^{B:W}}$ (Neutral substances) $P^{s:B} = \frac{0.037 \cdot 0.3 \cdot D_{OW} + 0.012 \cdot 2.0 \cdot D_{OW}^{0.94} + 0.259 \cdot 2.9 \cdot D_{OW}^{0.63} + 0.692}{P^{B:W}}$ (Ionic substances)	[5]
BCF_p	mL/g fish	Partitioning-based estimated bioconcentration factor	$P^{fish:B} = \frac{\frac{\sum_{i=1}^{9}(f_{nl,i} \cdot V_i)}{BM} \cdot K_{OW} + \frac{\sum_{i=1}^{9}(f_{pl,i} \cdot V_i)}{BM} \cdot K_{OW}^{0.94} + \frac{\sum_{i=1}^{9}(f_{nlom,i} \cdot V_i)}{BM} \cdot K_{OW}^{0.63} + \frac{\sum_{i=1}^{9}(f_{w,i} \cdot V_i)}{BM}}{P^{B:W}}$ (Neutral substances)	[5]

			$P^{fish:B} = \frac{\frac{\sum_{i=1}^{9}(f_{nl,i} \cdot V_i)}{BM} \cdot 0.3 \cdot D_{OW} + \frac{\sum_{i=1}^{9}(f_{pl,i} \cdot V_i)}{BM} \cdot 2.0 \cdot D_{OW}^{0.94} + \frac{\sum_{i=1}^{9}(f_{nlom,i} \cdot V_i)}{BM} \cdot 2.9 \cdot D_{OW}^{0.63} + \frac{\sum_{i=1}^{9}(f_{w,i}}{BM}}{P^{B:W}}$ (Ionic substances)	
γ_{water}	$L/kg^{0.75}/d$	Gill ventilation coefficient	$\gamma_{water} = \frac{VO_2}{0.71 \cdot C^{ox}} \cdot \frac{1}{1000^{0.25} \cdot M^{0.75}}$	[6]
γ_{blood}	$L/kg^{0.75}/d$	Blood perfusion coefficient	$\gamma_{blood} = F_{card} \cdot P^{B:W} \cdot \frac{1}{1000^{0.25} \cdot M^{0.75}}$	[1]
k_x	ml/d	Exchange coefficient between blood and water	$k_x = \frac{(\frac{M}{1000})^{0.75}}{2.8 \cdot 10^{-3} + \frac{68}{D_{OW,gill}} + \frac{1}{\gamma_{water}} + \frac{1}{\gamma_{blood}}} \cdot 1000$	[7]
k_u	d^{-1}	Assimilation rate constant from food	$k_u = \frac{0.8}{1 - 0.8} \cdot \frac{1}{0.03 \cdot (\frac{M}{1000})^{0.04} \cdot (D_{OW} - 1) + 1} \cdot \frac{(\frac{M}{1000})^{-0.25}}{1.1 \cdot 10^{-5} + \frac{68}{D_{OW}} + \frac{1}{0.03 \cdot (\frac{M}{1000})^{0.04} \cdot D_{OW} \cdot (1 - 0.8) \cdot 0.005}}$	[7]
f_{abs}	-	Fraction assimilated of food	$f_{abs} = \frac{0.8}{(1 - 0.8) \cdot 0.005} \cdot \frac{1}{0.03 \cdot (\frac{M}{1000})^{0.04} \cdot (D_{OW} - 1) + 1} \cdot \frac{1}{1.1 \cdot 10^{-5} + \frac{68}{D_{OW}} + \frac{1}{0.03 \cdot (\frac{M}{1000})^{0.04} \cdot D_{OW} \cdot (1 - 0.8) \cdot}}$	[7]
$k_{e\ feces}$	d^{-1}	Elimination rate constant of feces	$k_{e\ feces} = \frac{1}{0.03 \cdot (\frac{M}{1000})^{0.04} \cdot (D_{OW} - 1) + 1} \cdot \frac{(\frac{M}{1000})^{-0.25}}{1.1 \cdot 10^{-5} + \frac{68}{D_{OW}} + \frac{1}{0.03 \cdot (\frac{M}{1000})^{0.04} \cdot D_{OW} \cdot (1 - 0.8) \cdot 0.005}}$	[7]
$D_{plasma:w}$	-	Plasma:water distribution ratio	$D_{plasma:w} = 10^{0.75 \cdot K_{OW,ion} + 0.58}$	[8]

UF	-	Unbound fraction in blood	$UF = \frac{1}{1 + D_{plasma:w}}$	Present study
$k_{M,N}$	d^{-1}	Whole-body primary biotransformation rate constant normalised to a 10-g fish at 15 ℃ (288 K)	$k_{M,N} = \frac{\ln(2)}{HL_N}$	[9]
$k_{M,X}$	d^{-1}	Whole-body primary biotransformation rate constant corrected for body mass and temperature differences	$k_{M,X} = k_{M,N} \times (\frac{M}{10})^{-0.25} \times e^{-874 \times (\frac{1}{T} - \frac{1}{288})}$	[9]
V_D	ml blood/g fish	Apparent volume of distribution	$V^D \approx \frac{BCF_P}{P^{B:W}} = P^{fish:B}$	[10]
$Cl_{hepatic}$	ml blood/d/g fish	hepatic clearance	$Cl_{hepatic} = k_{M,X} \cdot V_D$	[11]
Q_i (t)	µg	Quantity in organ *i*	Equation S1-S7	-
C_i (t)	µg/ml	Concentration in organ *i*	$C_i = \frac{Q_i}{V_i}$	-

a- Compartments index organs: B = blood, f = adipose tissue, art = arterial blood, br = brain, git = gastrointestinal tract, go = gonads, k = kidney, l = liver, pp = poorly perfused tissue, rp = richly perfused tissue, s = skin and ven = venous blood.

b- As shown in Hendriks et al. 2005, the estimated regression constant (0.04) showed lower potential for polar substances to accumulate in neutral lipids when compared to the measurement (0.1-0.5). Therefore, we used 0.3 (average of the measurement) to avoid the underestimation of partition of polar chemicals into neutral lipid.

Model equations

Chemical quantity in brain, gonads, richly perfused tissue (RPT), poorly perfused tissue (PPT), adipose tissues, and skin:

$$\frac{dQ_i}{dt} = F_i \times \left(C_{art} - \frac{Q_i}{V_i \times P_{i:B}}\right) \tag{S1}$$

Chemical quantity in gastrointestinal tract:

$$\frac{dQ_{lumen\_git}}{dt} = f_{abs} \times Q_{ingest} - (k_u + k_{e\,feces}) \times Q_{lumen_{git}} \tag{S2}$$

$$\frac{dQ_{git}}{dt} = k_u \times Q_{lumen\_git} + F_{git} \times (C_{art} - \frac{Q_{git}}{V_{git} \times P_{git:B}}) \tag{S3}$$

Intrinsic clearance (Cl_{int}, mL blood/d/g fish) is traditionally estimated by running the existing *in vitro-in vivo* extrapolation models [10, 12] in reverse. However, due to highly uncertain biotransformation data and the expected $Q_l \gg Cl_{int}$, Cl_{int} was assumed the same value as hepatic clearance ($Cl_{hepatic}$, mL blood/d/g fish) in the present study. Metabolism in the liver was described as a linear (first-order) process. Consequently, chemical quantity in liver:

$$\frac{dQ_l}{dt} = F_l \times (C_{art} - \frac{Q_l}{V_l \times P_{l:B}}) + F_{rp} \times (\frac{Q_{rp}}{V_{rp} \times P_{rp:B}} - \frac{Q_l}{V_l \times P_{l:B}}) + F_{git} \times (\frac{Q_{git}}{V_{git} \times P_{git:B}} - \frac{Q_l}{V_l \times P_{l:B}}) + F_{go} \times (\frac{Q_{go}}{V_{go} \times P_{go:B}} - \frac{Q_l}{V_l \times P_{l:B}}) - \mathrm{Cl}_{hepatic} \times M \times \frac{Q_l}{V_l \times P_{l:B}} \tag{S4}$$

Chemical quantity in kidney:

$$\frac{dQ_k}{dt} = F_k \times (C_{art} - \frac{Q_k}{V_k \times P_{k:B}}) + (1 - \alpha_{Fpp}) \times F_{pp} \times (\frac{Q_{pp}}{V_{pp} \times P_{pp:B}} - \frac{Q_k}{V_k \times P_{k:B}}) + (1 - \alpha_{Fs}) \times F_s \times (\frac{Q_s}{V_s \times P_{s:B}} - \frac{Q_k}{V_k \times P_{k:B}}) \tag{S5}$$

Chemical concentration in venous blood:

$$C_{ven} = \frac{\sum_{i=1}^{6}(F_i \times \frac{Q_i}{V_i \times P_{i:B}})}{F_{card}} \tag{S6}$$

Chemical concentration in arterial blood:

$$C_{art} = C_{ven} + \frac{k_x \times (\mathrm{C_{water}} - \frac{C_{ven} \times \mathrm{UF}}{P_{B:w}})}{F_{card}} \tag{S7}$$

Section S2 Physiological data collection and treatment

Organ volumes

Organ volume values were obtained from FishBase [13] and literature (Figure S1). The volume of RPT was calculated by summing measured weights of spleen, heart, adrenal and thyroid in [14]. Arterial and venous blood was assumed to account for 1/3 and 2/3 of the total blood volume, respectively [15].

Tissue composition

Lipid and water contents of fish tissue (expressed as a percentage of tissue volume) were obtained from literature [16-22] consisiting of 22 fish species: *Anguilla Anguilla, Carassius auratus, Channa argus, Clupea harengus, Cyprinus carpio, Danio rerio, Esox Lucius, Gadus morhua, Ictalurus punctatus, Oncorhynchus keta, Oncorhynchus kisutch, Oncorhynchus mykiss, Oryzias laticeps, Perca fluviatilis, Pimephales promelas, Platichthys flesus, Poecilia reticulata, Rutilus rutilus, Salmo salar, Salvelinus namaycush, Scomber scombrus* and *Tilapia nilotica.*

Metabolic rates

Cardiac output values were obtained through literature search on Google Scholar with the search string 'fish cardiac output temperature'. Oxygen consumption rate values were derived from FishBase [13].

Weighting factor

The weighting factor was calculated as the ratio between tissue blood flow as a percentage of cardiac output and tissue volume as a percentage of body weight, based on values measured in rainbow trout from [23] and [24].

Section S3 Evaluation data search and filtering procedures

Evaluation data were obtained through an extensive literature search on Google Scholar. Search strings used in queries were related to pharmaceuticals (specific names) on one side, combined with terms related to toxicity (i.e. 'tissue distribution' and 'bioaccumulation') and 'fish' on the other. The first 50 hits published on or before 19 October 2020 (our cut-off for this study) were checked for relevance. The evaluation data met the following criteria: (i) Exposure scenarios were known; (ii) measurements covered a times series of internal concentrations; and (iii) concentrations were measured in either whole fish body or more than two organs.

Section S4 Comparison of internal concentrations of diclofenac and ibuprofen using partitioning estimation approaches outlined in Hendriks et al. [5] and Armitage et al. [25]

In our paper, tissue *i*: water partition coefficients ($P_{i:W}$) was calculated as [5]:

$$P_{i:W} = f_{nl} \cdot 0.3 \cdot D_{OW} + f_{pl} \cdot 2.0 \cdot D_{OW}^{0.94} + f_{nlom} \cdot 2.9 \cdot D_{OW}^{0.63} + f_w \tag{S8}$$

where D_{OW} was calculated at biological pH (i.e. 7.4 in whole fish body); f_{nl}, f_{pl}, f_{nlom} and f_w are the fractions of neutral lipid, polar lipid, non-lipid organic matter and water in respective tissues, respectively (Table 1 in the main text). In Armitage et al. [25], $P_{i:W}$ was calculated as:

$$P_{i:W} = f_{nl} \cdot D_{OW} + f_{pl} \cdot D_{MW} + f_{nlom} \cdot 0.05 \cdot D_{OW} + f_w$$

(S9)

where D_{MW} was the membrane-water distribution coefficient, combining the neutral (K_{MW}) and the ionic ($K_{MW,ion}$) molecules' contributions. The D_{MW} was calculated at biological pH (i.e. 7.4 in whole fish body) following the approach outlined in Armitage et al. [25]. Using available data [26], for diclofenac, D_{OW} (pH=7.4) = 90.2 and D_{MW} (pH=7.4) = 453. For ibuprofen, D_{OW} (pH=7.4) = 15.2 and D_{MW} (pH=7.4) = 70.9.

Results for diclofenac are shown below (left side using eq.(S8) right side using eq.(S9)):

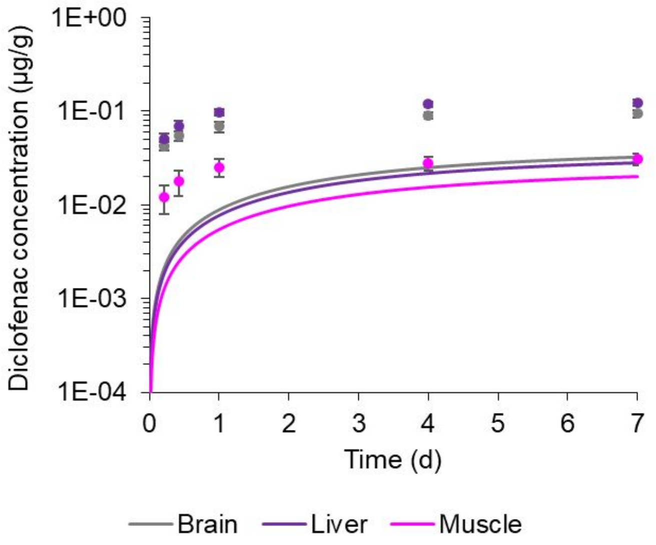

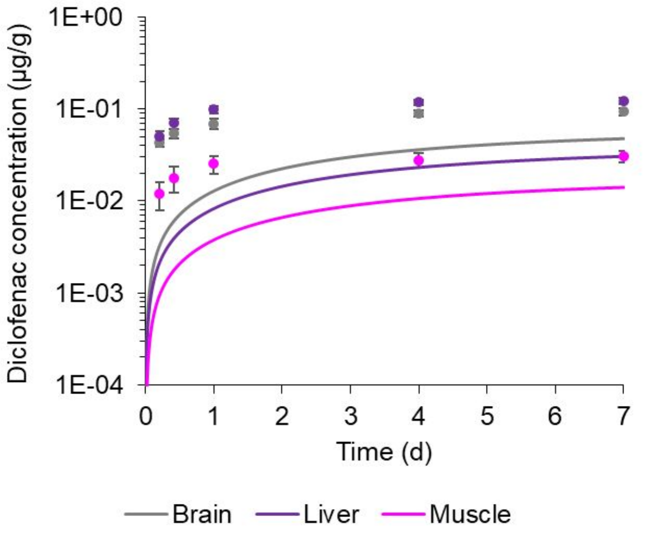

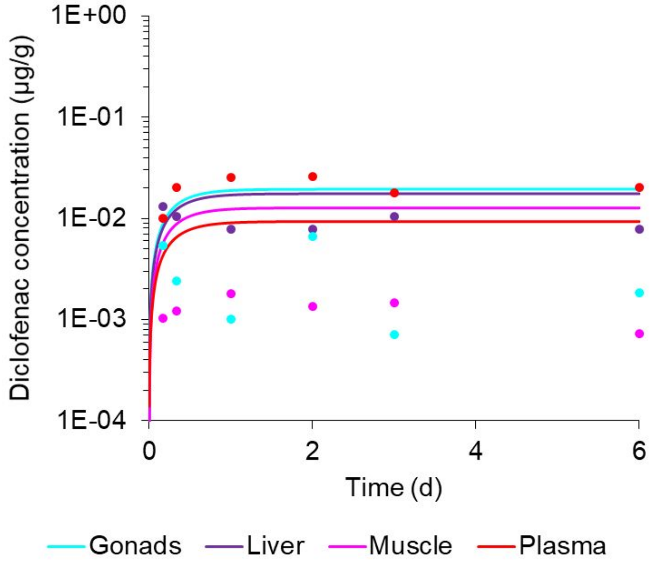

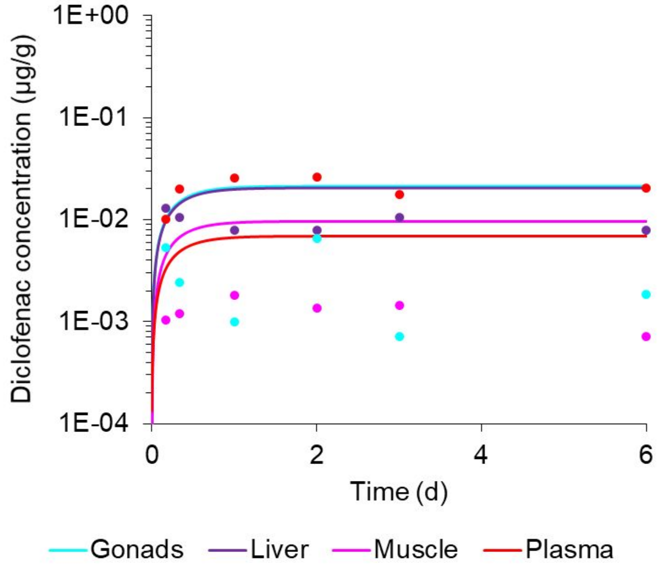

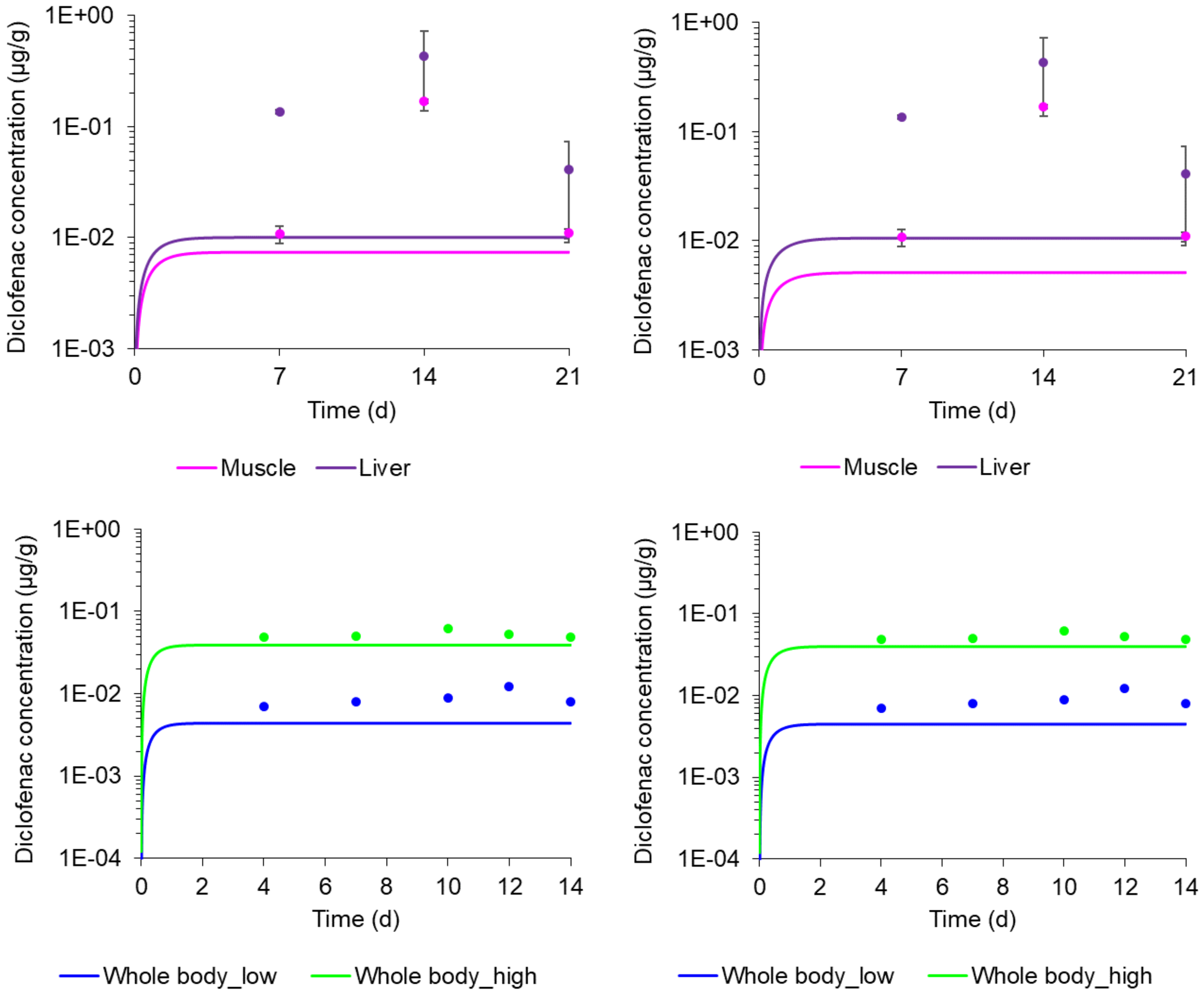

Results for ibuprofen are shown below (left side using eq.(S8) right side using eq.(S9)):

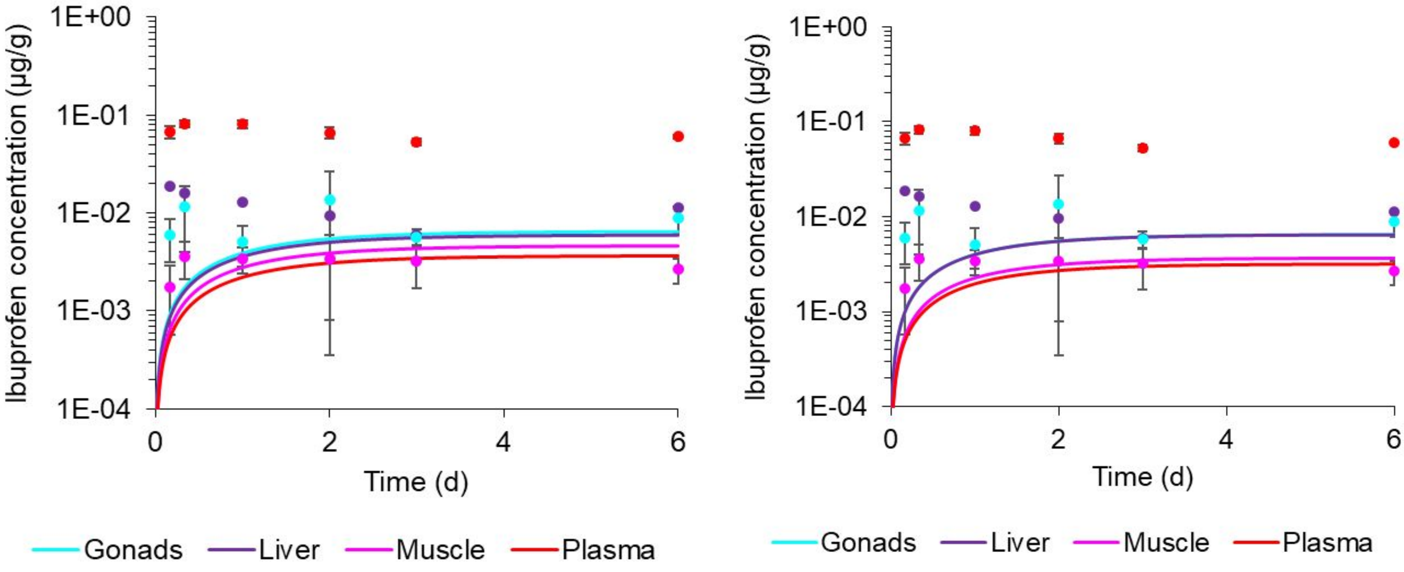

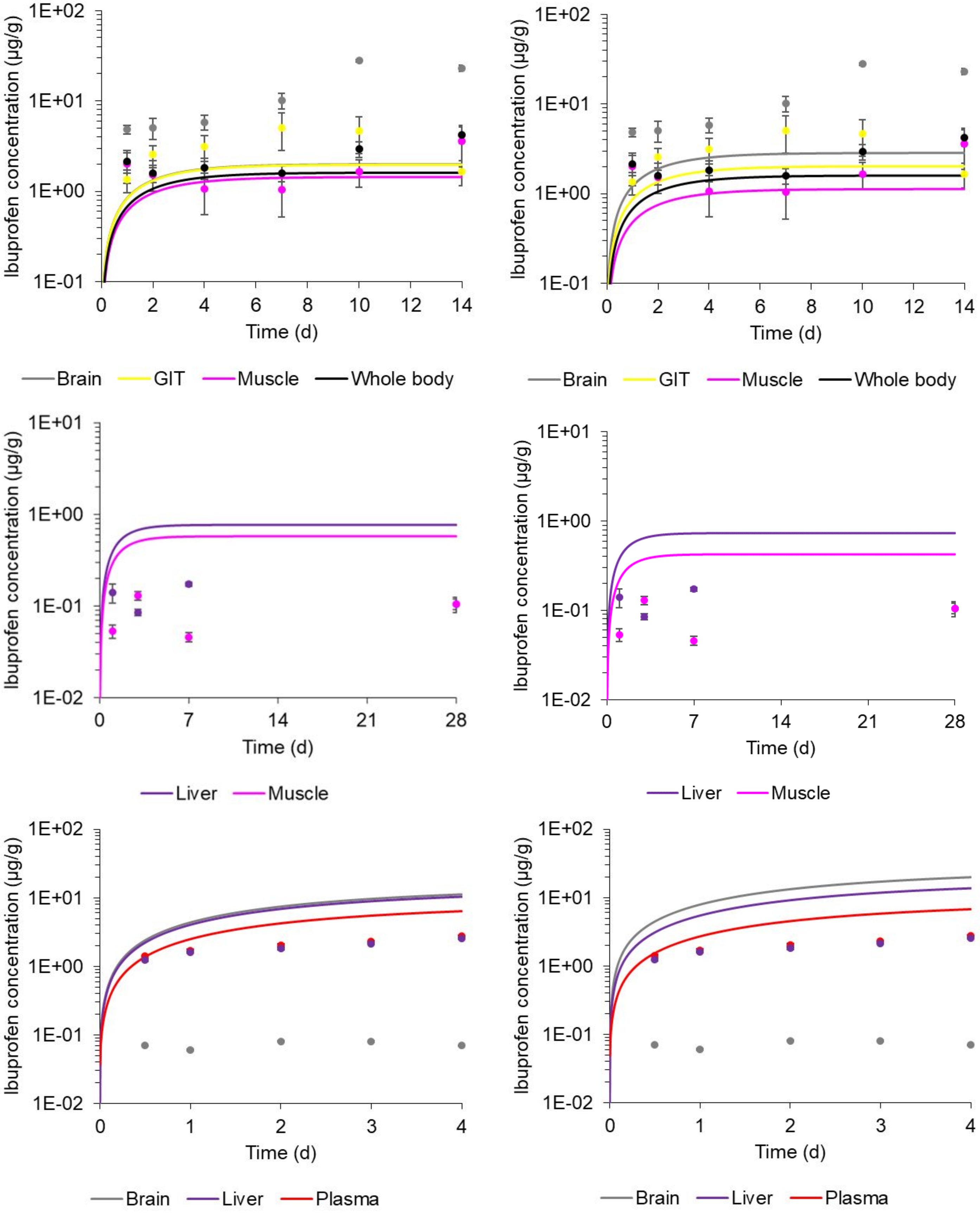
Ibuprofen concentration (µg/g)
Time (d)
Brain
GIT
Muscle
Whole body
Liver
Plasma
1E+02
1E+01
1E+00
1E-01
1E-02

For both sets of results, we only observed marginal differences in bioaccumulation, as the difference of tissue:blood partitioning was less than 2.2 times for all tissues including the whole body except for adipose tissue (5 times difference).

Chemical	Tissue	Tissue:blood partitioning using eq.(S8)	Tissue:blood partitioning using eq.(S9)
Diclofenac	Adipose fat	3.2	15.7
	Brain	2.2	4.8
	Kidney	1.9	3.3
	Liver	1.9	3.1
	Gastrointestinal tract	2.1	3.2
	Gonads	2.1	3.1
	Poorly perfused tissues	1.4	1.5
	Richly perfused tissues	2.3	2.2
	Skin	1.9	2.0
	Whole body	1.6	2.1
Ibuprofen	Adipose fat	1.6	9.0
	Brain	1.8	2.9
	Kidney	1.6	2.2
	Liver	1.6	2.0
	Gastrointestinal tract	1.7	2.1
	Gonads	1.8	2.0
	Poorly perfused tissues	1.3	1.2
	Richly perfused tissues	2.0	1.5
	Skin	1.7	1.5
	Whole body	1.4	1.8

Figures

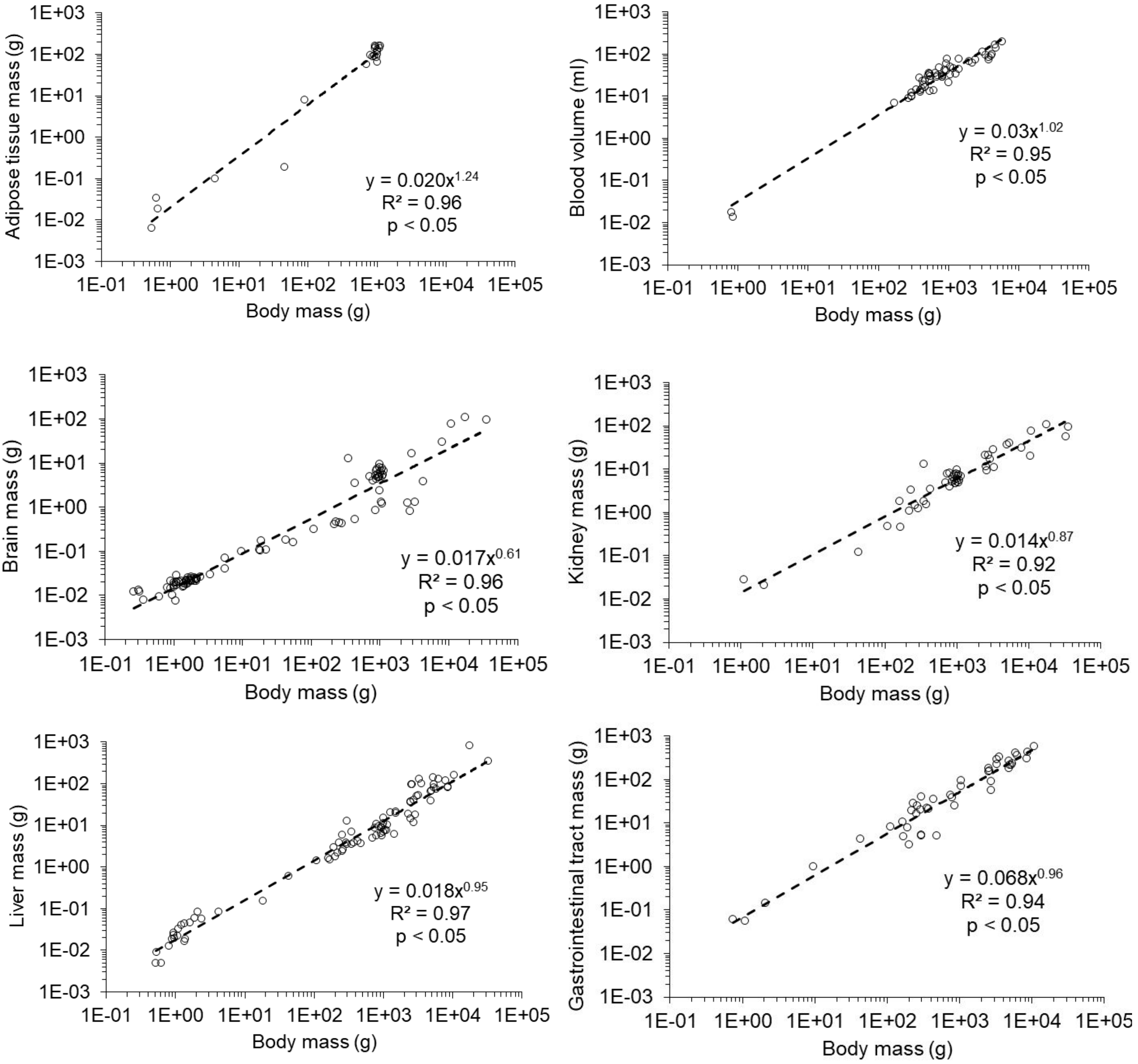
Adipose tissue mass (g)
Body mass (g)
y = 0.020x^1.24
R² = 0.96
p < 0.05
Blood volume (ml)
Body mass (g)
y = 0.03x^1.02
R² = 0.95
p < 0.05
Brain mass (g)
Body mass (g)
y = 0.017x^0.61
R² = 0.96
p < 0.05
Kidney mass (g)
Body mass (g)
y = 0.014x^0.87
R² = 0.92
p < 0.05
Liver mass (g)
Body mass (g)
y = 0.018x^0.95
R² = 0.97
p < 0.05
Gastrointestinal tract mass (g)
Body mass (g)
y = 0.068x^0.96
R² = 0.94
p < 0.05
1E-03
1E-02
1E-01
1E+00
1E+01
1E+02
1E+03
1E+04
1E+05

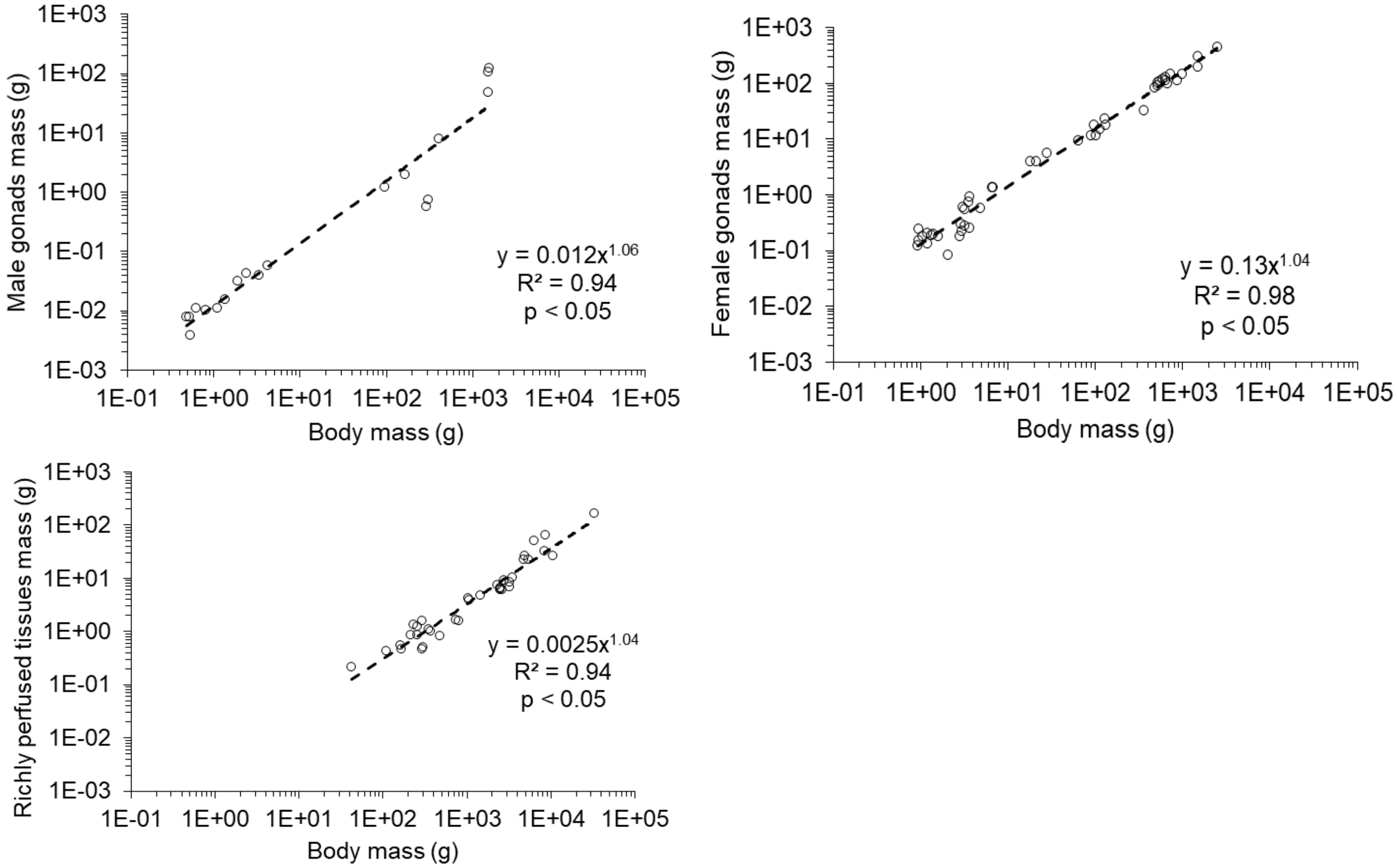

Figure S1 Fish organ/tissue mass (g) relative to fish body mass (g) based on literature. Data were retrieved from:

Adipose tissue mass-[1, 17, 18, 27-32];

Blood volume-[15, 23, 33-48];

Brain mass-[1, 13, 15, 21, 33, 49-52];

Kidney mass-[1, 14, 17-19, 33, 36];

Liver mass-[14, 15, 17-19, 21, 33, 36, 50, 51, 53-61];

Gastrointestinal tract mass-[2, 19, 33, 62-65];

Male gonads mass-[15, 19, 51, 53-55, 57, 59-61, 66-70];

Female gonads mass-[15, 19, 51, 53-55, 57, 59, 60, 66-74];

Richly perfused tissues mass-[14].

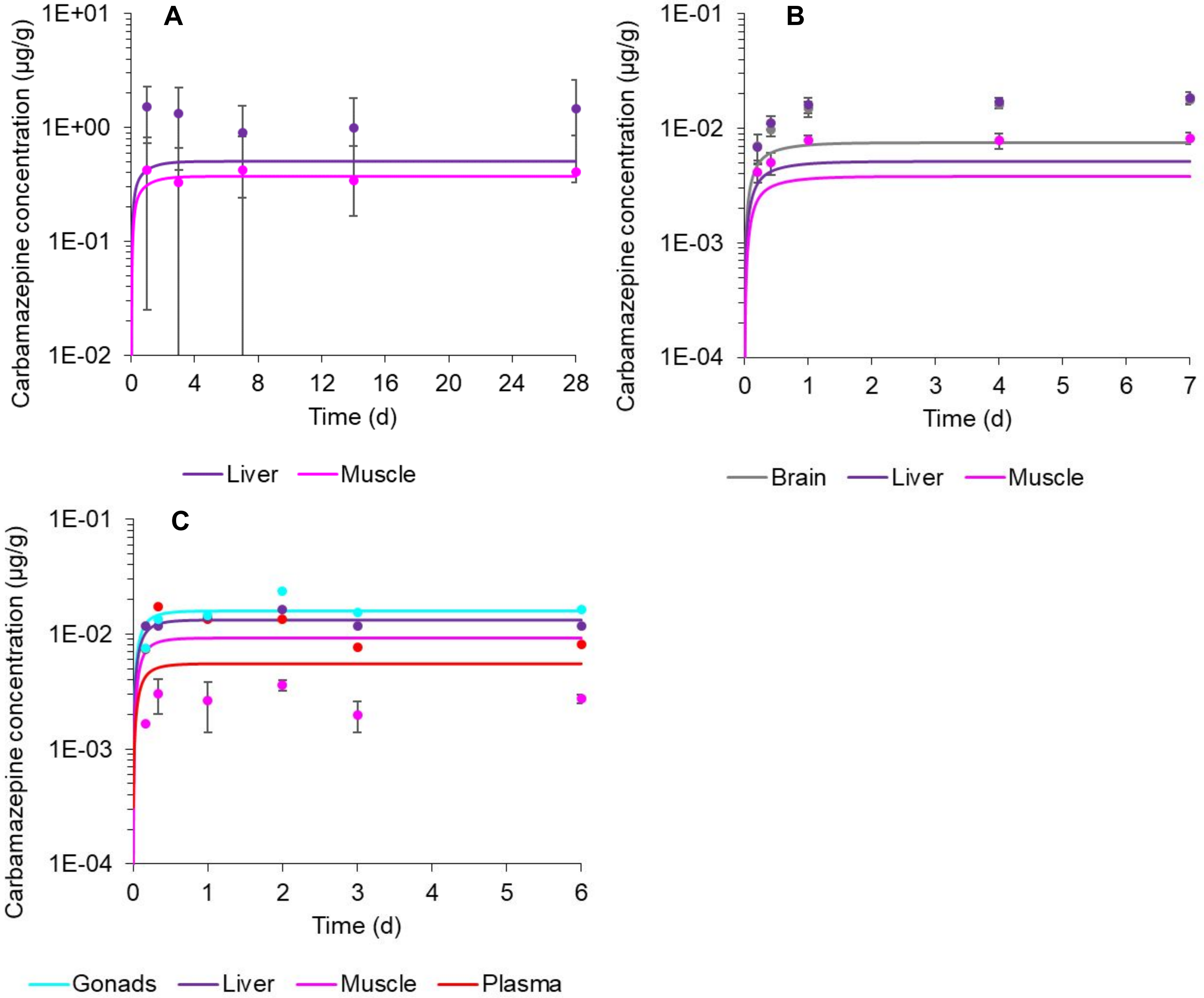

Figure S2 Time series of estimated carbamazepine concentrations (solid curves, µg/g) in organs (classified by colours) and measured concentrations (dots, µg/g) from literature in Pimephales notatus (A) [75], Carassius carassius (B) [76] and Danio rerio (C) [77]. The estimated concentrations were based on the biotransformation half-life derived from EPI Suite™ v4.11. Means and standard errors are shown.

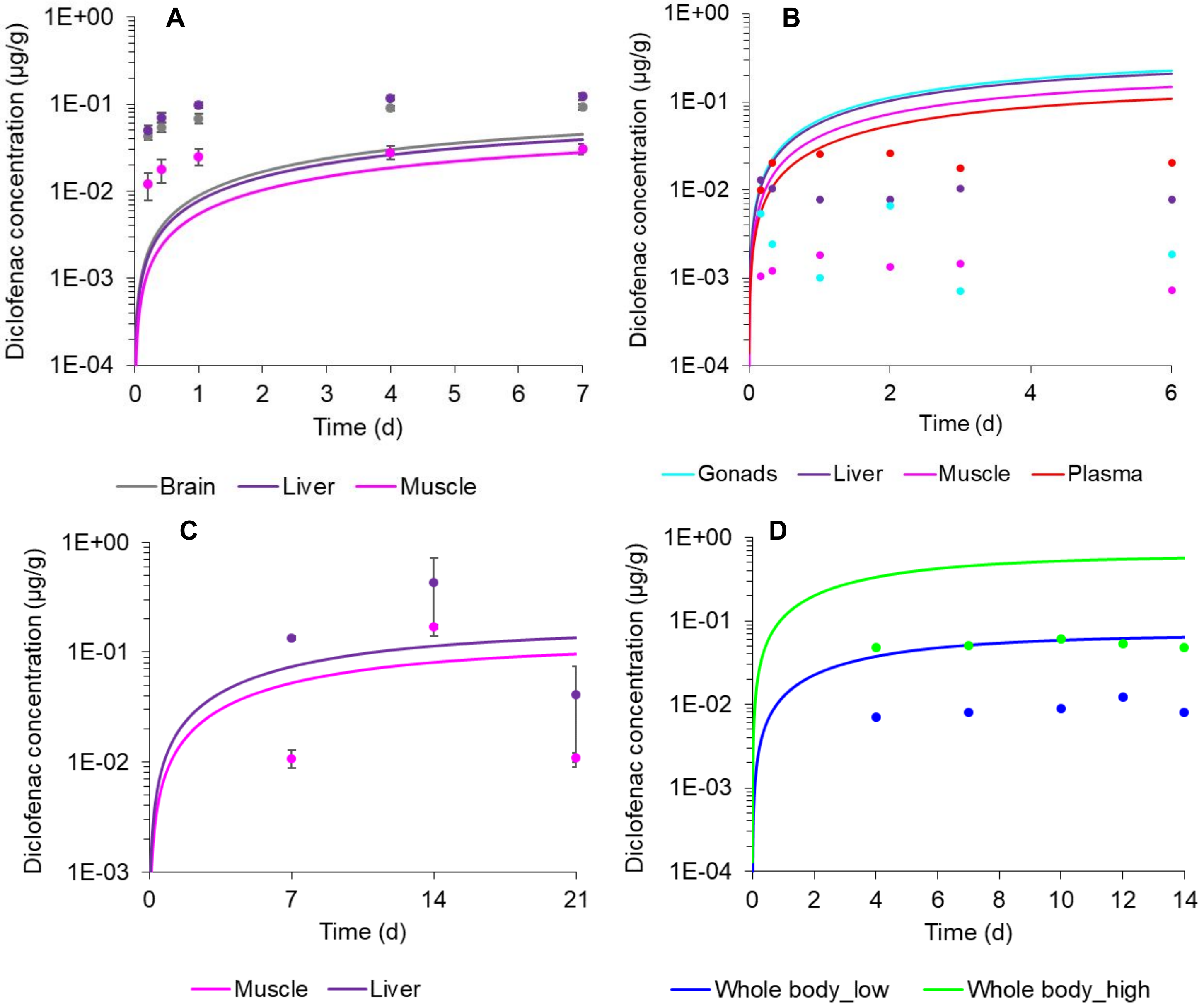

Figure S3 Time series of estimated diclofenac concentrations (solid curves, µg/g) in organs (classified by colours) and measured concentrations (dots, µg/g) from literature in Carassius carassius (A) [76], Danio rerio (B) [77], Carassius auratus (C) [78] and Oncorhynchus mykiss (D) [79]. The estimated concentrations were based on the biotransformation half-life derived from EPI Suite™ v4.11. Means and standard errors are shown.

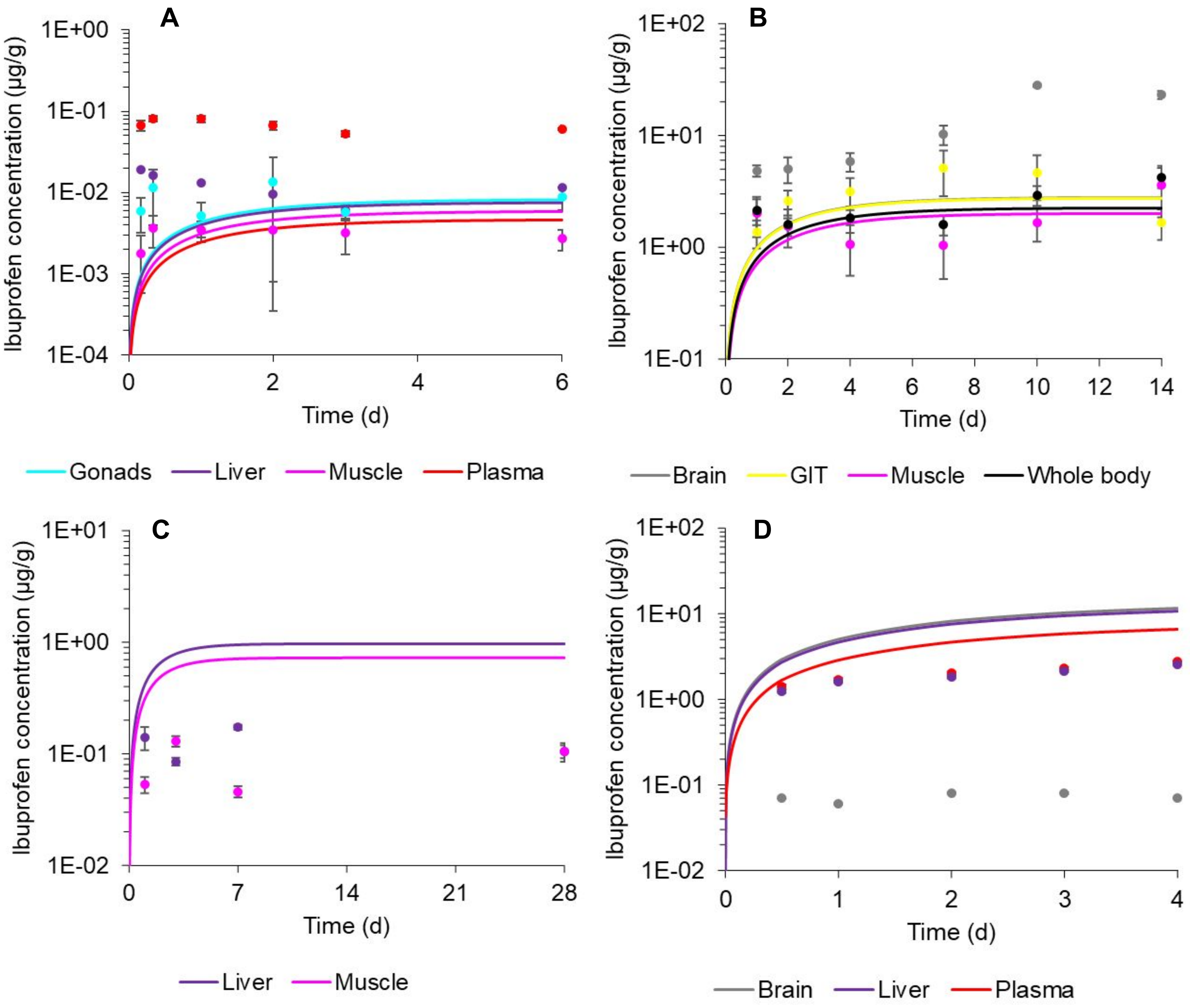

Figure S4 Times series of estimated ibuprofen concentrations (solid curves, μg/g) in organs (classified by colours) and measured concentrations (dots, μg/g) from literature in Danio rerio (A) [77], Cyprinus carpio (B) [80], Pimephales promelas (C) [81] and Cyprinus carpio (D) [82]. The estimated concentrations were based on the biotransformation half-life derived from EPI Suite™ v4.11. Means and standard errors are shown.

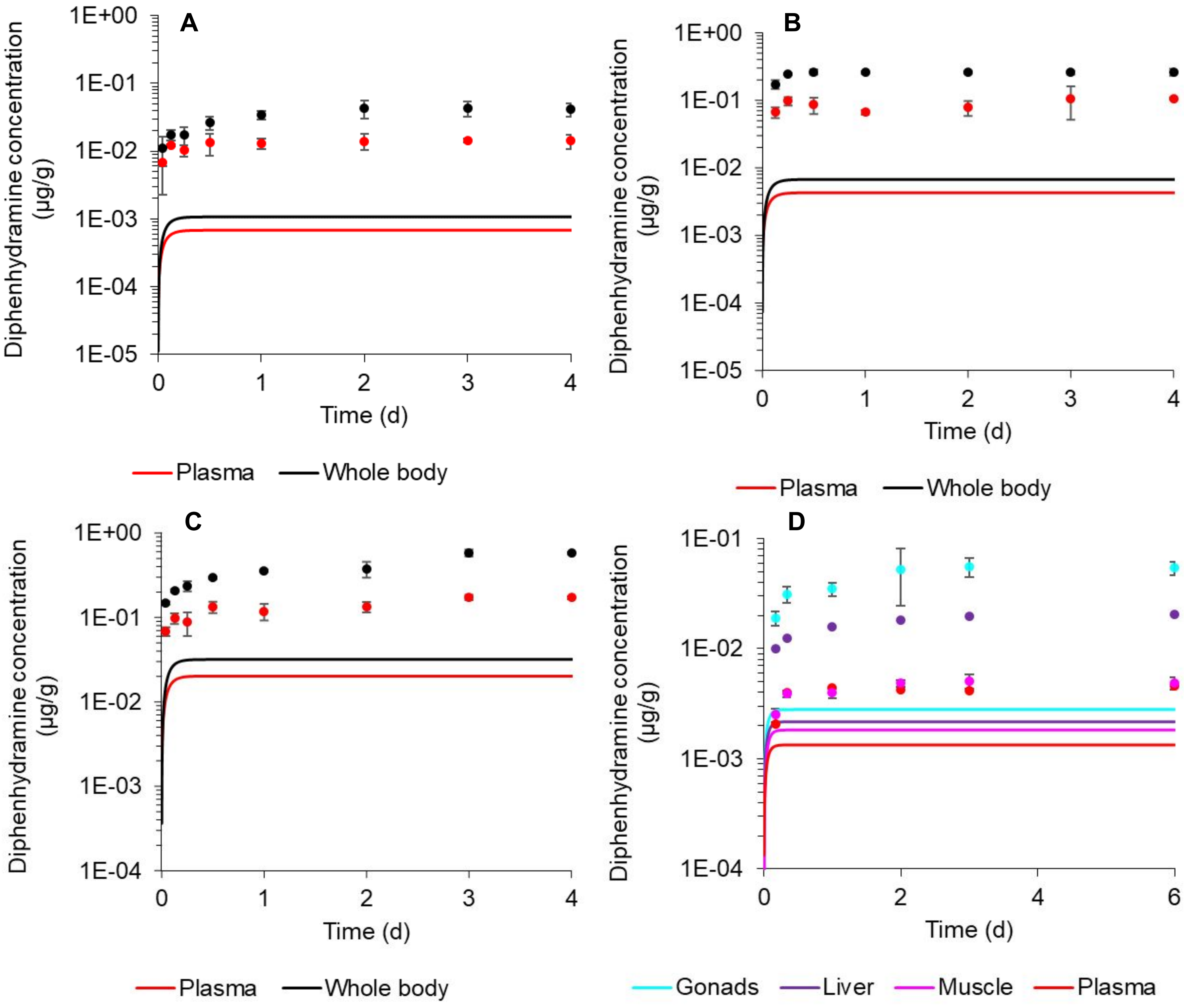

Figure S5 Times series of estimated diphenhydramine concentrations (solid curves, µg/g) in organs (classified by colours) and measured concentrations (dots, µg/g) from literature in Pimephales promelas (water pH=6.7) (A) [83], Pimephales promelas (water pH=7.7) (B) [83], Pimephales promelas (water pH=8.7) (C) [83] and Danio rerio (D) [77]. The estimated concentrations were based on the biotransformation half-life derived from EPI Suite™ v4.11. Means and standard errors are shown.

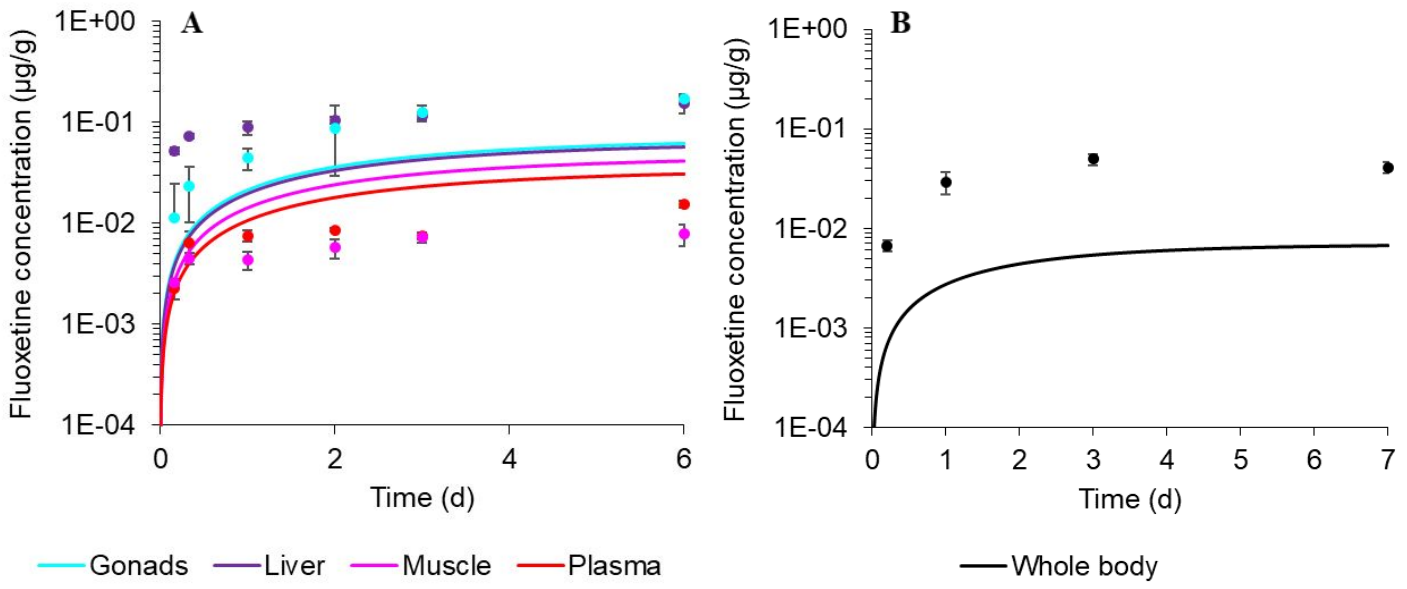

Figure S6 Times series of estimated fluoxetine concentrations (solid curves, µg/g) in organs (classified by colours) and measured concentrations (dots, µg/g) from literature in Danio rerio (A) [77] and Oryzias latipes (B) [84]. The estimated concentrations were based on the biotransformation half-life derived from EPI Suite™ v4.11. Means and standard errors are shown.

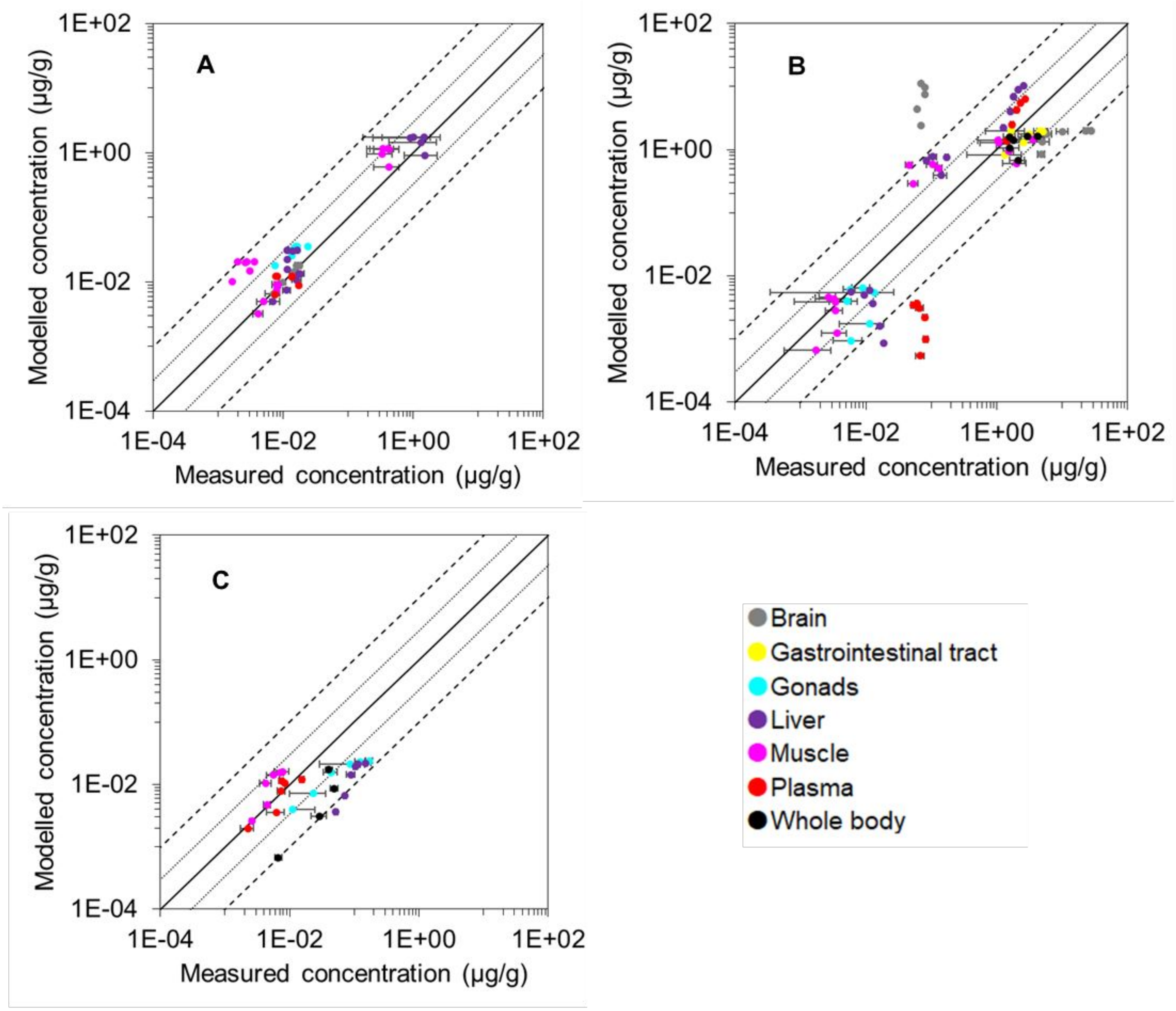

Figure S7 Comparison of modelled concentrations (µg/g) with measured concentrations (µg/g) for (A) carbamazepine, (B) ibuprofen and (C) fluoxetine. Modelled concentrations were based on the measured biotransformation half-life from literature. Organs are classified by colours. Means and standard errors are shown. Dotted and dashed lines represent the 3-fold and 10-fold changes, respectively.

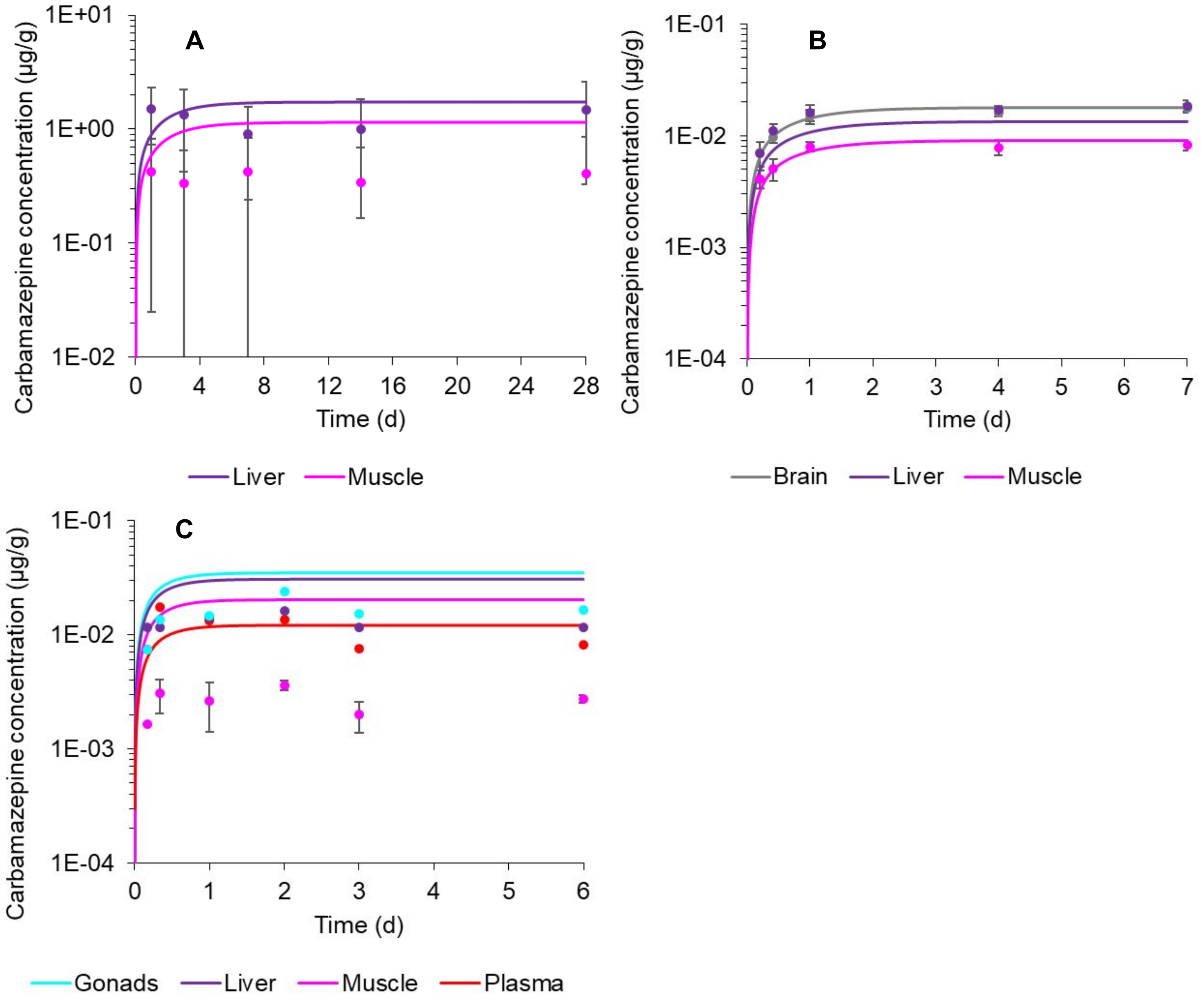

Figure S8 Times series of estimated carbamazepine concentrations (solid curves, μg/g) in organs (classified by colours) and measured concentrations (dots, μg/g) from literature in Pimephales notatus (A) [75], Carassius carassius (B) [76] and Danio rerio (C) [77]. The estimated concentrations were based on the measured biotransformation half-life in [77]. Means and standard errors are shown.

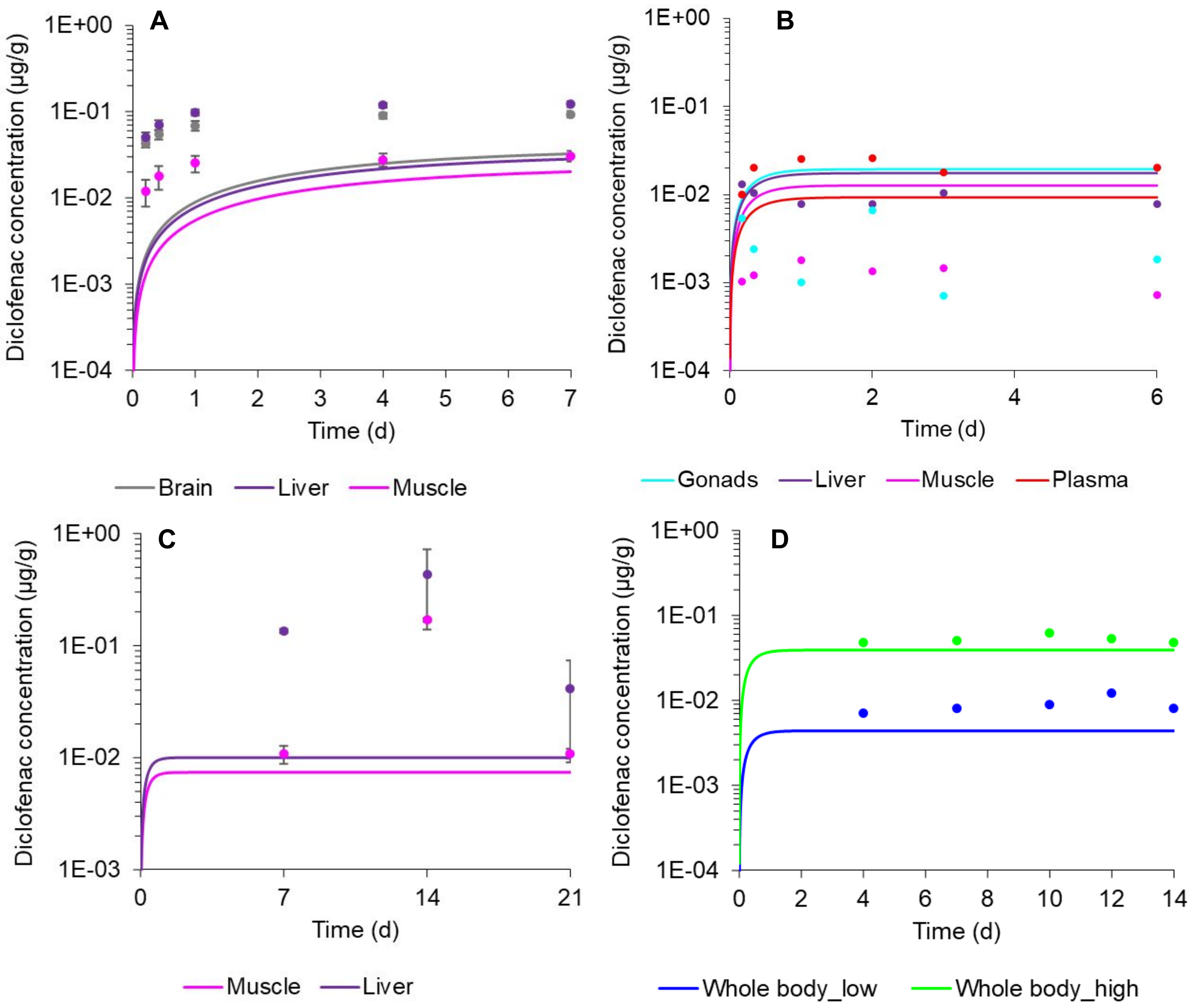

Figure S9 Times series of estimated diclofenac concentrations (solid curves, µg/g) in organs (classified by colours) and measured concentrations (dots, µg/g) from literature in Carassius carassius (A) [76], Danio rerio (B) [77], Carassius auratus (C) [78] and Oncorhynchus mykiss (D) [79]. The estimated concentrations were based on the measured biotransformation half-life in [77] and [76]. Means and standard errors are shown.

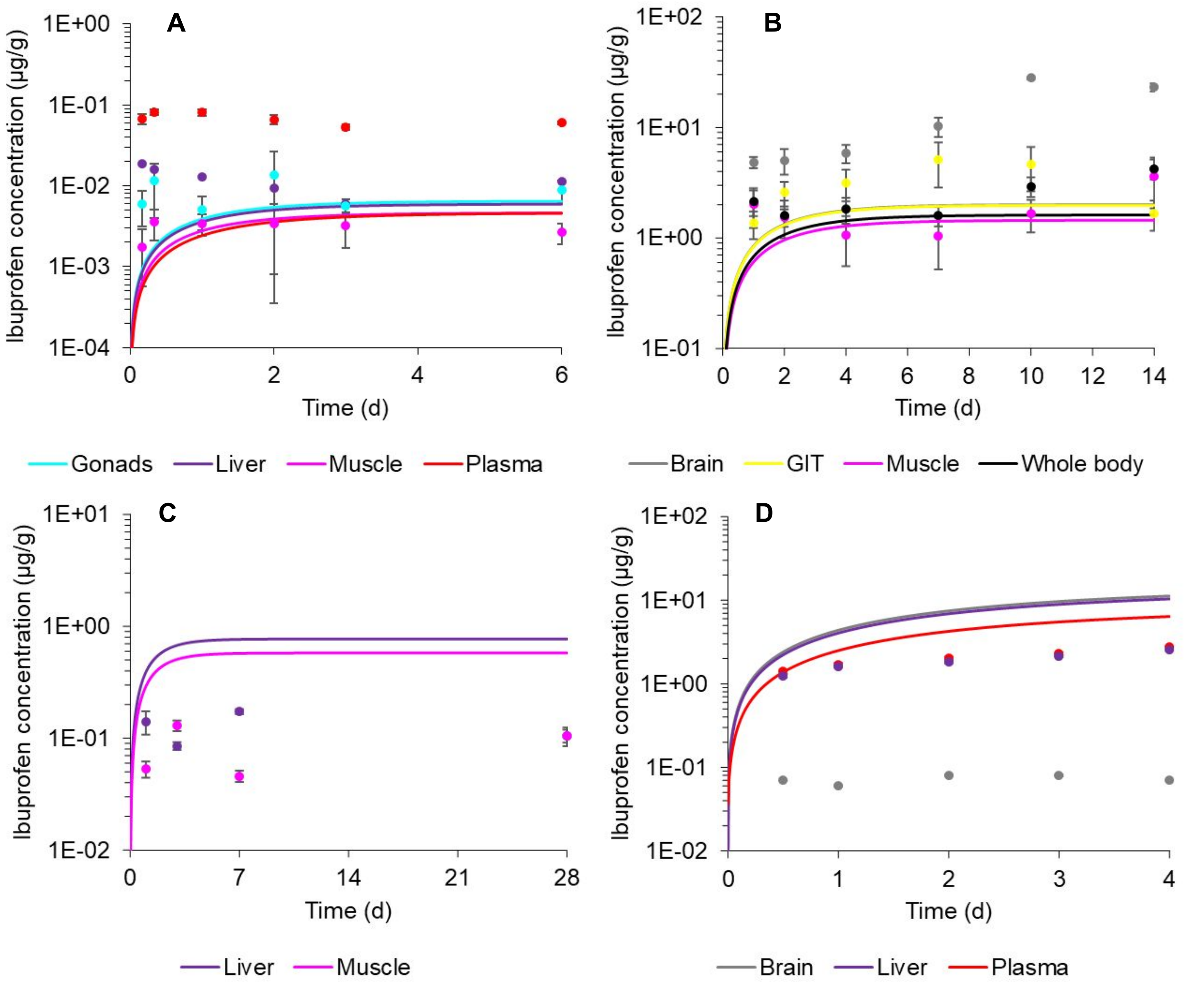

Figure S10 Times series of estimated ibuprofen concentrations (solid curves, µg/g) in organs (classified by colours) and measured concentrations (dots, µg/g) from literature in Danio rerio (A) [77], Cyprinus carpio (B) [80], Pimephales promelas (C) [81] and Cyprinus carpio (D) [82]. The estimated concentrations were based on the measured biotransformation half-life in [77]. Means and standard errors are shown.

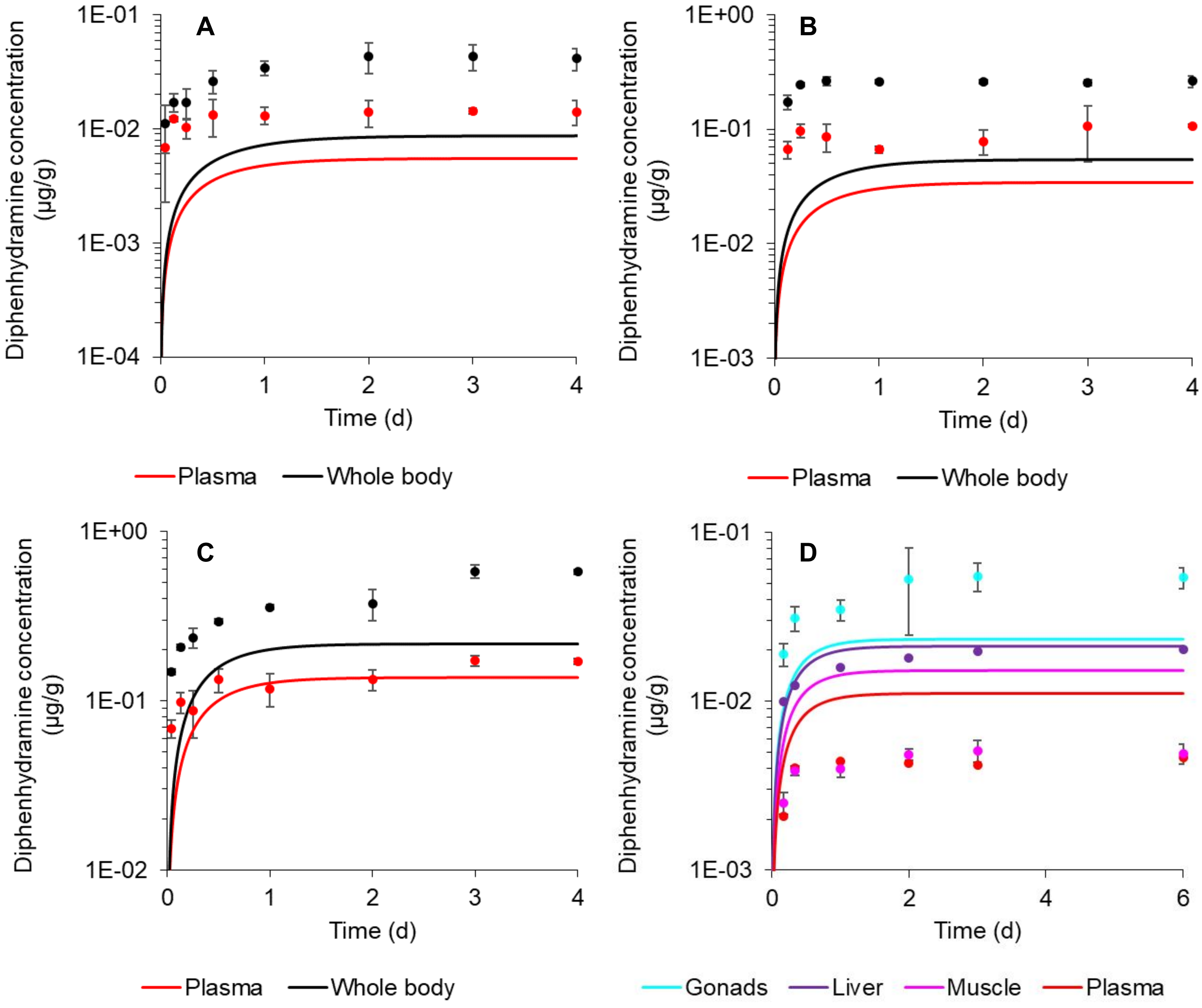

Figure S11 Times series of estimated diphenhydramine concentrations (solid curves, µg/g) in organs (classified by colours) and measured concentrations (dots, µg/g) from literature in Pimephales promelas (water pH=6.7) (A) [83], Pimephales promelas (water pH=7.7) (B) [83], Pimephales promelas (water pH=8.7) (C) [83] and Danio rerio (D) [77]. The estimated concentrations were based on the measured biotransformation half-life in [77]. Means and standard errors are shown.

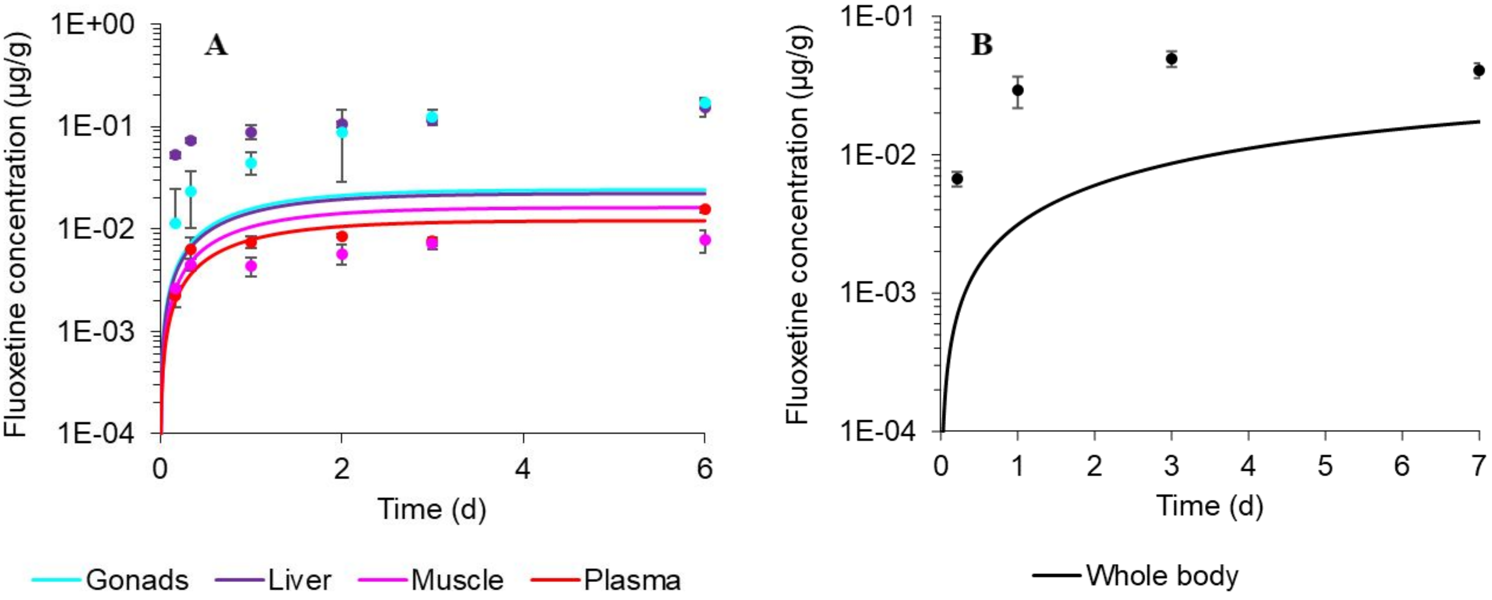

Figure S12 Times series of estimated fluoxetine concentrations (solid curves, µg/g) in organs (classified by colours) and measured concentrations (dots, µg/g) from literature in Danio rerio (A) [77] and Oryzias latipes (B) [84]. The estimated concentrations were based on the measured biotransformation half-life in [77] and [84]. Means and standard errors are shown.

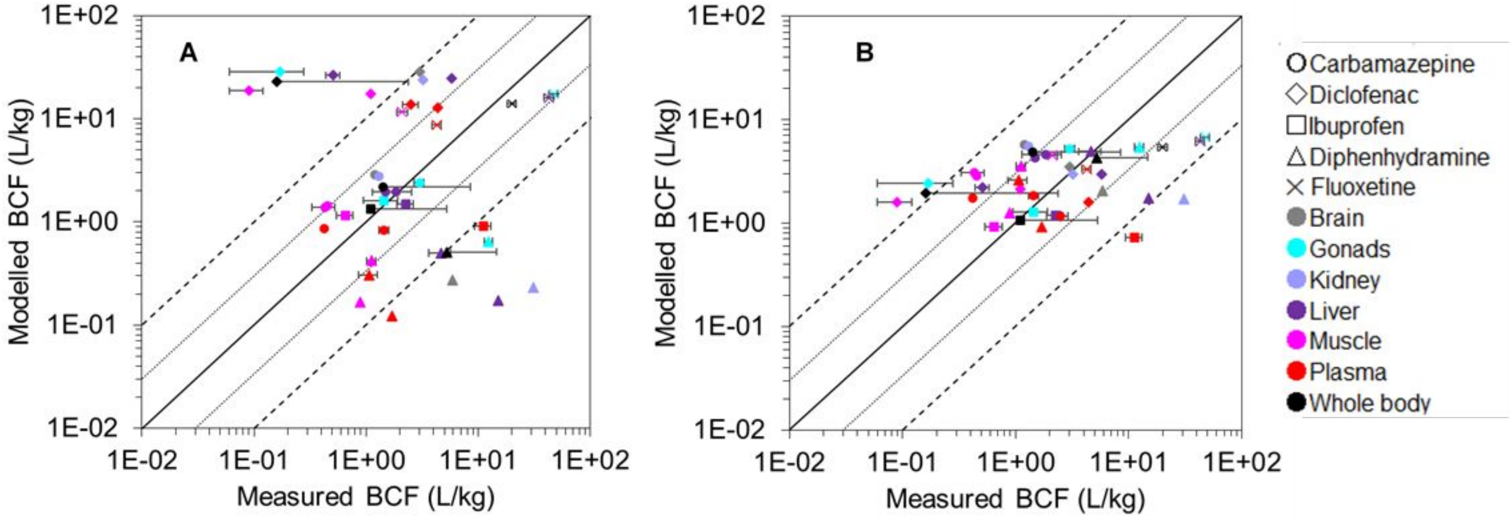

Figure S13 Comparison of modelled bioconcentration factors (BCF, L/kg) with measured BCFs (L/kg) from literature [77, 85]. Modelled BCFs were based on the biotransformation half-life from EPI Suite™ v4.11 [9] (A) and [77] (B), respectively. Organs and pharmaceuticals are classified by colours and shapes, respectively. Means and standard errors are shown. Dotted and dashed lines represent the 3-fold and 10-fold changes, respectively.

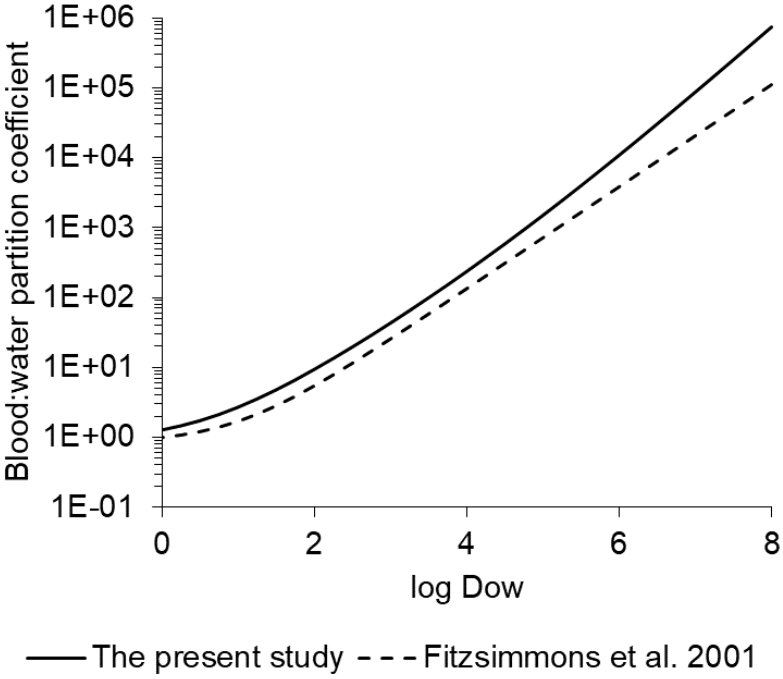

Figure S14 Comparison of estimated blood:water partition coefficient from the present study and [86].

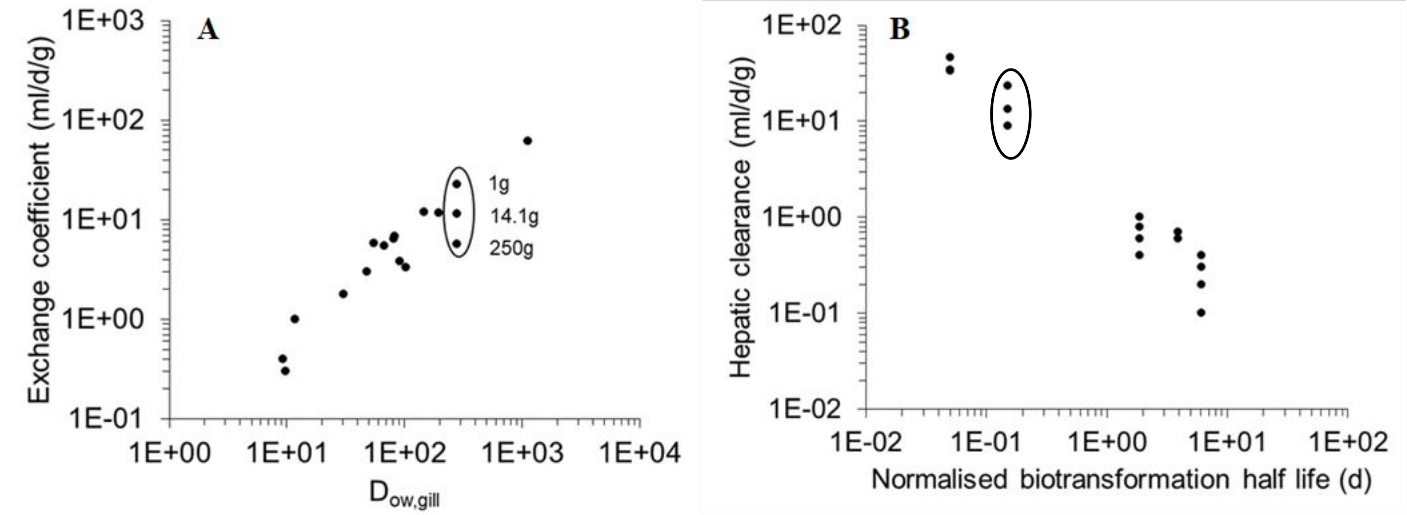

Figure S15 (A) Exchange coefficient (mL/d/g) versus the octanol-water distribution coefficient at the gill surface and fish body mass. (B) Hepatic clearance (mL/d/g) versus normalised biotransformation half-life (d) and fish body mass. The circled data represent fish body mass of 1g, 14.1g and 250g.

Tables

Table S2 Regression summary of organ/tissue volumes related to fish body mass (M, g)[a]

Parameter	Abbreviation	$N_{species}$	Regression with 95% CI	R^2	P-value
Adipose fat volume	V_f (ml)	7	$V_f = 0.020[0.010, 0.040] \cdot M^{1.24[1.13, 1.36]}$	0.96	3.80E-15
Blood volume	V_{bl} (ml)	25	$V_{bl} = 0.030[0.021, 0.049] \cdot M^{1.02[0.96, 1.08]}$	0.96	2.38E-41
Brain volume	V_{br} (ml)	13	$V_b = 0.017[0.015, 0.018] \cdot M^{0.61[0.58, 0.64]}$	0.96	1.31E-46
Kidney volume	V_k (ml)	22	$V_k = 0.014[0.008, 0.025] \cdot M^{0.87[0.80, 0.95]}$	0.92	1.78E-26
Liver volume	V_l (ml)	39	$V_l = 0.018[0.014, 0.023] \cdot M^{0.95[0.91, 1.00]}$	0.97	7.18E-66
Gastrointestinal tract volume	V_{git} (ml)	27	$V_{git} = 0.068[0.041, 0.113] \cdot M^{0.96[0.89, 1.03]}$	0.94	5.18E-28
Gonads volume (male)	$V_{go,m}$ (ml)	10	$V_{go,m} = 0.012[0.007, 0.021] \cdot M^{1.06[0.92, 1.20}$	0.94	1.27E-11
Gonads volume (female)	$V_{go,f}$ (ml)	8	$V_{go,f} = 0.13[0.11, 0.15] \cdot M^{1.04[0.99, 1.08]}$	0.98	2.38E-41
Richly perfused tissues volume	V_{rp} (ml)	25	$V_{rp} = 0.0025[0.0013, 0.0047] \cdot M^{1.04[0.95, 1.1}$	0.94	1.11E-22
Skin volume	V_s (ml)	-	$V_s = 0.10M^{0.67}$ [87]	-	-
Skeleton volume	V_{skel} (ml)	-	$V_{skel} = 0.033M^{1.03}$ [87]	-	-

Table S3 Regression summary of temperature-dependent parameters related to fish body mass (M, g) and/or absolute temperature (T, K)[a]

Parameter	Abbreviation	$N_{species}$	Regression with 95% CI	R^2	P-value
Cardiac output	F_{card} (ml/d)	17	$F_{card} = e^{25.5[21.9, 29.1]} \cdot e^{\frac{-5790[-6812, -4770]}{T}} \cdot$ M	0.56	2.39E-19
Oxygen consumption rate	VO_2 (mg O_2/d)	67	$VO_2 = e^{19.1[16.5, 21.6]} \cdot e^{\frac{-5040[-5775, -4312]}{T}} \cdot$ M	0.42	7.21E-32
Dissolved oxygen concentration	C_{ox} (mg O_2/ml)	-	$C_{ox} = e^{-10.6[-10.7, -10.5]} \cdot e^{\frac{1743.3[1717, 1769]}{T}}$	0.99	1.20E-53

[a] Data were retrieved from: Cardiac output-[6, 23, 24, 88-111]; Oxygen consumption rate-[13]; Dissolved oxygen concentration-[112].

Table S4 Physicochemical properties of selected pharmaceuticals in this study[a]

Compound	Abbr.	Formula	Chemical class	MW (g/mol)	pK_a[b]	$\log K_{OW}$[c]	$\log K_{OW,ion}$[d]	Biotransformation HL (d)[c]
Carbamazepine	CBZ	$C_{15}H_{12}N_2O$	Neutral	236.3	-	2.45	-	0.15
Diclofenac	DCF	$C_{14}H_{11}Cl_2NO_2$	Acid	296.1	4.18	4.51	1.85	6.13
Diphenhydramine	DPH	$C_{17}H_{21}NO$	Base	255.4	8.98	3.56[e]	1.70	0.05
Fluoxetine	FXT	$C_{17}H_{18}F_3NO$	Base	309.3	10.1	4.05	1.51	3.89

Ibuprofen	IBU	$C_{13}H_{18}O_2$	Acid	206.3	4.41	3.97	0.75	1.88

[a] MW, molecular weight; log K_{ow}, logarithm of octanol-water partition coefficient.

[b] Values were obtained from SCIfinder.

[c] Values were obtained from the experimental database in EPI Suite™ v4.11.

[d] Values were obtained from Molinspiration (http://www.molinspiration.com//cgi-bin/properties).

[e] [83].

Table S5 Validation data as a result an extensive literature search. Abbreviation: CBZ=carbamazepine, DCF=diclofenac, IBU=ibuprofen, DPH=diphenhydramine, FXT=fluoxetine

Chemical	Species	Sex/Age	Fish weight (g)	Water temp. (°C)	Water pH	Exposure route	Exposure concentration (µg/mL)	Exposure duration	Organs	Ref.
CBZ	Pimephales notatus	Adult	250	25 ± 2	8.0 ± 2.2	Aqueous	0.3	28d	Liver, muscle	[75]
CBZ	Carassius carassius	Juvenile	14.1 ±2.17	21 ± 2	7.38 ± 0.70	Aqueous	0.00225	7d	Liver, muscle, brain	[76]
CBZ	Danio rerio	Female/Adult	1	25 ± 2	7.6	Aqueous	0.00535	6d	Plasma, liver, gonads, muscle	[77]
DCF	Carassius carassius	Juvenile	14.1 ± 2.17	21 ± 2	7.38 ± 0.70	Aqueous	0.00175	7d	Liver, muscle, brain	[76]
DCF	Carassius carassius	Adult	40.5 ± 3.6	20 ± 1	7.2 ± 0.2	Aqueous	0.00357	21d	Liver, muscle	[78]
DCF	Oncorhynchus mykiss	Juvenile	1.1	13.5-15.0	7.5-8.4	Aqueous	0.0021, 0.0187	14d	Whole body	[79]
DCF	Danio rerio	Female/Adult	1	25 ± 2	7.6	Aqueous	0.008	6d	Plasma, liver, gonads, muscle	[77]
DPH	Pimephales promelas	Male/Adult	3.27	25 ± 1	6.7, 7.7, 8.7	Aqueous	0.0093	96h	Whole body, plasma	[83]
DPH	Danio rerio	Female/Adult	1	25 ± 2	7.6	Aqueous	0.00435	6d	Plasma, liver, gonads, muscle	[77]
FXT	Danio rerio	Female/Adult	1	25 ± 2	7.6	Aqueous	0.0036	6d	Plasma, liver, gonads, muscle	[77]
FXT	Oryzias latipes	Adult	0.36 ± 0.02	25 ± 2	7.4	Aqueous	0.00055	7d	Whole body	[84]
IBU	Pimephales promelas	Adult	3	20	6.5	Aqueous	0.1526	28d	Liver, muscle	[81]
IBU	Cyprinus carpio	Adult	9.1 ± 0.5	25 ± 2	7.8 ± 0.2	Aqueous	1.92	14d	GIT, muscle, brain, whole body	[80]
IBU	Cyprinus carpio	Juvenile	50.7 ± 7.8	20 ± 2	7.5-8.0	Aqueous	17.56	96h	Blood, liver, brain	[82]
IBU	Danio rerio	Female/Adult	1	25 ± 2	7.6	Aqueous	0.00504	6d	Plasma, liver, gonads, muscle	[77]

Table S6 % of predictions within 10- and 3-fold differences (number of measurements).

Set I	Carbamazepine		Diclofenac		Ibuprofen		Diphenhydramine		Fluoxetine	
	FD<10	FD <3	FD <10	FC<3	FD <10	FD <3	FD <10	FD <3	FD <10	FD <3
Brain	100 (5)	100 (5)	60 (5)	40 (5)	45 (11)	9 (11)				
GIT					100 (6)	100 (6)				
Gonads	100 (6)	100 (6)	33 (6)	17 (6)	100 (6)	67 (6)	17 (6)	0 (6)	100 (6)	100 (6)
Liver	100 (16)	75 (16)	57 (14)	21 (14)	93 (15)	33 (15)	100 (6)	0 (6)	83 (6)	33 (6)
Muscle	94 (16)	75 (16)	57 (14)	21 (14)	100 (16)	73 (16)	100 (6)	100 (6)	100 (6)	33 (6)
Plasma	100 (6)	83 (6)	100 (6)	67 (6)	45 (11)	18 (11)	48 (29)	3 (29)	100 (6)	83 (6)
Whole body			80 (10)	0 (10)	100 (6)	100 (6)	17 (23)	0 (23)	75 (4)	0 (4)
All	100 (49)	82 (49)	64 (55)	24 (55)	80 (71)	50 (71)	44 (70)	10 (70)	93 (28)	54 (28)

Set II	Carbamazepine		Diclofenac		Ibuprofen		Diphenhydramine		Fluoxetine	
	FD <10	FD <3	FD <10	FD <3	FD <10	FD <3	FD <10	FD <3	FD <10	FD <3
Brain	100 (5)	100(5)	60 (5)	20 (5)	36 (11)	0 (11)				
GIT					100 (6)	100 (6)				
Gonads	100 (6)	100 (6)	50 (6)	33 (6)	100 (6)	67 (6)	100 (6)	100 (6)	100 (6)	33 (6)
Liver	100 (16)	100 (16)	64 (14)	43 (14)	87 (15)	40 (15)	100 (6)	100 (6)	67 (6)	0 (6)
Muscle	94 (16)	56 (16)	85 (14)	29 (14)	100 (16)	73 (16)	100 (6)	50 (6)	100 (6)	100 (6)
Plasma	100 (6)	100 (6)	100 (6)	83 (6)	45 (11)	45 (11)	97 (29)	66 (29)	100 (6)	100 (6)
Whole body			100 (10)	100 (10)	100 (6)	83 (6)	86 (23)	26 (23)	100 (4)	25 (4)
All	98 (49)	86 (49)	78 (55)	51 (55)	77 (71)	52 (71)	94 (70)	57 (70)	93 (28)	54 (28)

	Set I		Set II	
	FD <10	FD <3	FD <10	FD <3
Brain	62 (21)	38 (21)	57 (21)	29 (21)
GIT	100 (6)	100 (6)	100 (6)	100 (6)
Gonads	70 (30)	57 (30)	90 (30)	67 (30)
Liver	86 (57)	39 (57)	84 (57)	60 (57)
Muscle	88 (58)	59 (58)	93 (58)	57 (58)
Plasma	64 (58)	29 (58)	88 (58)	71 (58)
Whole body	49 (43)	14 (43)	93 (43)	51 (43)

[a] GIT=gastrointestinal tract.

Table S7 Comparison of estimated and measured tissue-blood partition coefficients in [85]

Compound	Brain		Liver		Kidney		Muscle	
	This study	Measured (95%CI)	This study	Measured (95%CI)	This study	Measured (95%CI)	This study	Measured (95%CI)
Carbamazepine	3.3	3.2 (1.3-8.3)	2.6	3.9 (1.6-8.1)	3.2	3.4 (1.5-6.9)	1.8	1.1 (0.27-2.2)
Diclofenac	2.2	0.66 (0.3-1.1)	1.9	1.5 (0.19-3.2)	1.9	0.79 (0.11-2.0)	1.4	0.30 (0.07-0.76)
Diphenhydramine	2.2	4.1 (1.7-7.2)	1.9	12 (2.6-28)	1.9	21 (5.1-49)	1.4	0.63 (0.27-1.1)

Table S8 Summary of existing fish PBK models

Study	Chemical	Fish species	Model performance (N=data points)		
			FC<10	FC<5	FC<3
[113]	24 organic chemicals	Rainbow trout, fathead minnow	95%/88%[a] (N=39)	77%/80%[a] (N=68)	-
[15]	6 non-ionic organic chemicals	Zebrafish	-	88% (N=65)	-
[21]	24 neutral organic chemicals	Zebrafish	-	84% (N=31)	-
[19]	9 organic chemicals incl. ethinylestradiol and oxytetracycline	Rainbow trout, zebrafish, fathead minnow, stickleback	84%/78%/ 93%/76%[b]	-	57%/49%/ 65%/57%[b]
The present study	5 pharmaceuticals	-	73%/87%[c] (N=273)	54%/73%[c] (N=273)	41%/59%[c] (N=273)

[a] Results are for rainbow trout/fathead minnow.

[b] Results are for rainbow trout/zebrafish/fathead minnow/stickleback.

[c] Results are based on biotransformation half-lives from EPI Suite™ v4.11/experiments.

References

1. Nichols, J.W., J.M. McKim, M.E. Andersen, M.L. Gargas, H.J. Clewell, and R.J. Erickson. 1990. A physiologically based toxicokinetic model for the uptake and disposition of waterborne organic chemicals in fish. *Toxicology and Applied Pharmacology*, 106(3):433-447.
2. Nichols, J.W., J.M. McKim, G.J. Lien, A.D. Hoffman, S.L. Bertelsen, and C.M. Elonen. 1996. A physiologically based toxicokinetic model for dermal absorption of organic chemicals by fish. *Fundamental and Applied Toxicology*, 31(2):229-242.
3. Henderson, L.J. 1908. Concerning the relationship between the strength of acids and their capacity to preserve neutrality. *American Journal of Physiology-Legacy Content*, 21(2):173-179.
4. Rendal, C., K.O. Kusk, and S. Trapp. 2011. Optimal choice of pH for toxicity and bioaccumulation studies of ionizing organic chemicals. *Environmental Toxicology and Chemistry*, 30(11):2395-2406.
5. Hendriks, A.J., T.P. Traas, and M.A. Huijbregts. 2005. Critical body residues linked to octanol− water partitioning, organism composition, and LC50 QSARs: Meta-analysis and model. *Environmental Science & Technology*, 39(9):3226-3236.
6. Erickson, R.J. and J.M. McKim. 1990. A model for exchange of organic chemicals at fish gills: flow and diffusion limitations. *Aquatic Toxicology*, 18(4):175-197.
7. Hendriks, A.J., A. van der Linde, G. Cornelissen, and D.T.H.M. Sijm. 2001. The power of size. 1. Rate constants and equilibrium ratios for accumulation of organic substances related to octanol-water partition ratio and species weight. *Environmental Toxicology and Chemistry*, 20(7):1399-1420.
8. Nolte, T., Fate and effects of ionisable organic compounds and nanoparticles: At the interface of theory and experimentation. 2018, PhD thesis, Radboud University, Nijmegen, the Netherlands.
9. Arnot, J.A., W. Meylan, J. Tunkel, P.H. Howard, D. Mackay, M. Bonnell, and R.S. Boethling. 2009. A quantitative structure-activity relationship for predicting metabolic biotransformation rates for organic chemicals in fish. *Environmental Toxicology and Chemistry*, 28(6):1168-1177.
10. Nichols, J.W., I.R. Schultz, and P.N. Fitzsimmons. 2006. In vitro–in vivo extrapolation of quantitative hepatic biotransformation data for fish: I. A review of methods, and strategies for incorporating intrinsic clearance estimates into chemical kinetic models. *Aquatic Toxicology*, 78(1):74-90.

11. Papa, E., J.A. Arnot, A. Sangion, and P. Gramatica, In Silico Approaches for the Prediction of In Vivo Biotransformation Rates, in Advances in QSAR Modeling. 2017, Springer. p. 425-451.

12. Nichols, J.W., P.N. Fitzsimmons, and L.P. Burkhard. 2007. In vitro-in vivo extrapolation of quantitative hepatic biotransformation data for fish. II. Modeled effects on chemical bioaccumulation. *Environmental Toxicology and Chemistry*, 26(6):1304-1319.

13. Froese, R. and D. Pauly. *FishBase*. 2019; Available from: www.fishbase.org, version (12/2019).

14. Crile, G. and D.P. Quiring. 1940. A record of the body weight and certain organ and gland weights of 3690 animals.

15. Péry, A.R., J. Devillers, C. Brochot, E. Mombelli, O. Palluel, B. Piccini, F. Brion, and R. Beaudouin. 2014. A physiologically based toxicokinetic model for the zebrafish Danio rerio. *Environmental Science & Technology*, 48(1):781-790.

16. Bertelsen, S.L., A.D. Hoffman, C.A. Gallinat, C.M. Elonen, and J.W. Nichols. 1998. Evaluation of log KOW and tissue lipid content as predictors of chemical partitioning to fish tissues. *Environmental Toxicology and Chemistry*, 17(8):1447-1455.

17. Lien, G.J., J.M. McKim, A.D. Hoffman, and C.T. Jenson. 2001. A physiologically based toxicokinetic model for lake trout (Salvelinus namaycush). *Aquatic Toxicology*, 51(3):335-350.

18. Nichols, J.W., J. McKim, G. Lien, A. Hoffman, S. Bertelsen, and C. Gallinat. 1993. Physiologically-based toxicokinetic modeling of three waterborne chloroethanes in channel catfish, Ictalurus punctatus. *Aquatic Toxicology*, 27(1-2):83-111.

19. Grech, A., C. Tebby, C. Brochot, F.Y. Bois, A. Bado-Nilles, J.-L. Dorne, N. Quignot, and R. Beaudouin. 2019. Generic physiologically-based toxicokinetic modelling for fish: Integration of environmental factors and species variability. *Science of the Total Environment*, 651:516-531.

20. Henderson, R.J. and D.R. Tocher. 1987. The lipid composition and biochemistry of freshwater fish. *Progress in Lipid Research*, 26(4):281-347.

21. Brinkmann, M., C. Schlechtriem, M. Reininghaus, K. Eichbaum, S. Buchinger, G. Reifferscheid, H. Hollert, and T.G. Preuss. 2016. Cross-Species Extrapolation of Uptake and Disposition of Neutral Organic Chemicals in Fish Using a Multispecies Physiologically-Based Toxicokinetic Model Framework. *Environmental Science & Technology*, 50(4):1914-1923.

22. Ewald, G. and P. Larsson. 1994. Partitioning of 14c-labelled 2,2′,4,4′-tetrachlorobiphenyl between water and fish lipids. *Environmental Toxicology and Chemistry*, 13(10):1577-1580.

23. Barron, M., B. Tarr, and W. Hayton. 1987. Temperature-dependence of cardiac output and regional blood flow in rainbow trout, Salmo gairdneri Richardson. *Journal of Fish Biology*, 31(6):735-744.

24. Wilson, R. and S. Egginton. 1994. Assessment of maximum sustainable swimming performance in rainbow trout (Oncorhynchus mykiss). *Journal of Experimental Biology*, 192(1):299-305.

25. Armitage, J.M., J.A. Arnot, F. Wania, and D. Mackay. 2013. Development and evaluation of a mechanistic bioconcentration model for ionogenic organic chemicals in fish. *Environmental Toxicology and Chemistry*, 32(1):115-128.

26. Avdeef, A., K.J. Box, J.E.A. Comer, C. Hibbert, and K.Y. Tam. 1998. pH-Metric logP 10. Determination of Liposomal Membrane-Water Partition Coefficients of lonizable Drugs. *Pharmaceutical Research*, 15(2):209-215.

27. Lien, G.J., J.W. Nichols, J.M. McKim, and C.A. Gallinat. 1994. Modeling the accumulation of three waterborne chlorinated ethanes in fathead minnows (Pimephales promelas): A physiologically based approach. *Environmental Toxicology and Chemistry: An International Journal*, 13(7):1195-1205.

28. Black, C., R. Scott, and M. Bernards. 2014. Seasonal changes in carotenoid and lipid storage by threespine stickleback, Gasterosteus aculeatus. *Environmental biology of fishes*, 97(2):209-214.

29. Ali, M. and R.J. Wootton. 2001. Does timing of daily feeding affect growth rates of juvenile three-spined sticklebacks, Gasterosteus aculeatus L? *Ecology of Freshwater Fish*, 10(3):127-131.

30. Könemann, H. and K. van Leeuwen. 1980. Toxicokinetics in fish: accumulation and elimination of six chlorobenzenes by guppies. *Chemosphere*, 9(1):3-19.

31. Delahunty, G. and V. De Vlaming. 1980. Seasonal relationships of ovary weight, liver weight and fat stores with body weight in the goldfish, Carassius auratus (L.). *Journal of Fish Biology*, 16(1):5-13.

32. Jobling, M., J. Koskela, and R. Savolainen. 1998. Influence of dietary fat level and increased adiposity on growth and fat deposition in rainbow trout, Oncorhynchus mykiss (Walbaum). *Aquaculture Research*, 29(8):601-607.

33. Gingerich, W.H., R.A. Pityer, and J.J. Rach. 1990. Whole body and tissue blood volumes of two strains of rainbow trout (Oncorhynchus mykiss). *Comparative Biochemistry and Physiology Part A: Physiology*, 97(4):615-620.

34. Gingerich, W., R. Pityer, and J. Rach. 1987. Estimates of plasma, packed cell and total blood volume in tissues of the rainbow trout (Salmo gairdneri). *Comparative biochemistry and physiology. A, Comparative physiology*, 87(2):251-256.

35. Gingerich, W. and R. Pityer. 1989. Comparison of whole body and tissue blood volumes in rainbow trout (Salmo gairdneri) with 125 I bovine serum albumin and 51 Cr-erythrocyte tracers. *Fish physiology and biochemistry*, 6(1):39-47.

36. Zhang, S., Z. Wang, and J. Chen. 2019. Physiologically based toxicokinetics (PBTK) models for pharmaceuticals and personal care products in wild common carp (Cyprinus carpio). *Chemosphere*, 220:793-801.

37. Ronald, K., H.C. Macnab, J.E. Stewart, and B. Beaton. 1964. BLOOD PROPERTIES OF AQUATIC VERTEBRATES: I. TOTAL BLOOD VOLUME OF THE ATLANTIC COD, GADUS MORHUA L. *Canadian Journal of Zoology*, 42(6):1127-1132.

38. Skov, P.V. and J.F. Steffensen. 2003. The blood volumes of the primary and secondary circulatory system in the Atlantic cod Gadus morhua L., using plasma bound Evans Blue and compartmental analysis. *Journal of experimental biology*, 206(3):591-599.

39. Duff, D., D. Fitzgerald, D. Kullman, D. Lipke, J. Ward, and K. Olson. 1987. Blood volume and red cell space in tissues of the rainbow trout, Salmo gairdneri. *Comparative Biochemistry and Physiology Part A: Physiology*, 87(2):393-398.

40. Conte, F.P., H.H. Wagner, and T.O. Harris. 1963. Measurement of blood volume in the fish (Salmo gairdneri gairdneri). *American Journal of Physiology-Legacy Content*, 205(3):533-540.

41. Nikinmaa, M., A. Soivio, and E. Railo. 1981. Blood volume of Salmo gairdneri: Influence of ambient temperature. *Comparative Biochemistry and Physiology Part A: Physiology*, 69(4):767-769.

42. Nichols, D.J. 1987. Fluid volumes in rainbow trout, Salmo gairdneri: application of compartmental analysis. *Comparative biochemistry and physiology. A, Comparative physiology*, 87(3):703-709.

43. Smith, L.S. 1966. Blood volumes of three salmonids. *Journal of the Fisheries Board of Canada*, 23(9):1439-1446.

44. Brill, R., K. Cousins, D. Jones, P.G. Bushnell, and J.F. Steffensen. 1998. Blood volume, plasma volume and circulation time in a high-energy-demand teleost, the yellowfin tuna (Thunnus albacares). *The Journal of Experimental Biology*, 201(5):647-654.

45. Tort, L., F. González-Arch, P. Torres, and J. Hidalgo. 1991. On the blood volume of the Mediterranean dogfish, Scyliorhinus canicula. *Fish Physiology and Biochemistry*, 9(2):173-177.

46. Thorson, T.B. 1961. The partitioning of body water in Osteichthyes: phylogenetic and ecological implications in aquatic vertebrates. *The Biological Bulletin*, 120(2):238-254.

47. Sleet, R. and L. Weber. 1983. Blood volume of a marine teleost before and after arterial cannulation. *Comparative Biochemistry and Physiology Part A: Physiology*, 76(4):791-794.

48. Itazawa, Y., T. Takeda, K.-i. Yamamoto, and T. Azuma. 1983. Determination of circulating blood volume in three teleosts, carp, yellowtail and porgy. *Japanese Journal of Ichthyology*, 30(1):94-101.

49. Li, Z., K.J. Kroll, K.M. Jensen, D.L. Villeneuve, G.T. Ankley, J.V. Brian, M.S. Sepúlveda, E.F. Orlando, J.M. Lazorchak, and M. Kostich. 2011. A computational model of the hypothalamic-pituitary-gonadal axis in female fathead minnows (Pimephales promelas) exposed to 17α-ethynylestradiol and 17β-trenbolone. *BMC systems biology*, 5(1):63.

50. Breen, M., D.L. Villeneuve, G.T. Ankley, D.C. Bencic, M.S. Breen, K.H. Watanabe, A.L. Lloyd, and R.B. Conolly. 2013. Developing predictive approaches to characterize adaptive responses of the reproductive endocrine axis to aromatase inhibition: II. Computational modeling. *toxicological sciences*, 133(2):234-247.

51. Ji, K., S. Hong, Y. Kho, and K. Choi. 2013. Effects of bisphenol S exposure on endocrine functions and reproduction of zebrafish. *Environmental Science & Technology*, 47(15):8793-8800.

52. Gao, D., M. Wu, C. Wang, Y. Wang, and Z. Zuo. 2015. Chronic exposure to low benzo [a] pyrene level causes neurodegenerative disease-like syndromes in zebrafish (Danio rerio). *Aquatic toxicology*, 167:200-208.

53. Borton, D.L., D.L. Cook, W.K. Bradley, R.E. Philbeck, M.G. Dubé, N.J. Brown-Peterson, and W.R. Streblow. 2009. Responses of fathead minnows (pimephales promelas) during life-cycle exposures to pulp mill effluents at four long-term receiving water study sites. *Integrated Environmental Assessment and Management: An International Journal*, 5(2):270-282.

54. Sowers, A.D., K.M. Gaworecki, M.A. Mills, A.P. Roberts, and S.J. Klaine. 2009. Developmental effects of a municipal wastewater effluent on two generations of the fathead minnow, Pimephales promelas. *Aquatic toxicology*, 95(3):173-181.

55. Watanabe, K.H., K.M. Jensen, E.F. Orlando, and G.T. Ankley. 2007. What is normal? A characterization of the values and variability in reproductive endpoints of the fathead minnow, Pimephales promelas. *Comparative Biochemistry and Physiology Part C: Toxicology & Pharmacology*, 146(3):348-356.

56. Koivusaari, U. 1981. Seasonal variation of hepatic biotransformation in female and male rainbow trout (Salmo gairdneri).

57. Koivusaari, U. 1983. Thermal acclimatization of hepatic polysubstrate monooxygenase and UDP‐glucuronosyltransferase of mature rainbow trout (Salmo gairdneri). *Journal of Experimental Zoology*, 227(1):35-42.

58. Bon, E., U. Barbe, J.N. Rodriguez, B. Cuisset, C. Pelissero, J. Sumpter, and F. Le Menn. 1997. Plasma vitellogenin levels during the annual reproductive cycle of the female rainbow trout (Oncorhynchus mykiss): establishment and validation of an ELISA. *Comparative Biochemistry and Physiology Part B: Biochemistry and Molecular Biology*, 117(1):75-84.

59. Chang, J., S. Liu, S. Zhou, M. Wang, and G. Zhu. 2013. Effects of butachlor on reproduction and hormone levels in adult zebrafish (Danio rerio). *Experimental and Toxicologic Pathology*, 65(1-2):205-209.

60. Han, Z., S. Jiao, D. Kong, Z. Shan, and X. Zhang. 2011. Effects of β‐endosulfan on the growth and reproduction of zebrafish (Danio rerio). *Environmental Toxicology and Chemistry*, 30(11):2525-2531.

61. Velasco-Santamaría, Y.M., B. Korsgaard, S.S. Madsen, and P. Bjerregaard. 2011. Bezafibrate, a lipid-lowering pharmaceutical, as a potential endocrine disruptor in male zebrafish (Danio rerio). *Aquatic Toxicology*, 105(1-2):107-118.

62. Oosten, J.v. 1957. The skin and scales. *The Physiology of Fishes, Vol. 1*:207-244.

63. Stevens, E.D. 1968. The effect of exercise on the distribution of blood to various organs in rainbow trout. *Comparative biochemistry and physiology*, 25(2):615-625.

64. Varanasi, U., M. Uhler, and S.I. Stranahan. 1978. Uptake and release of naphthalene and its metabolites in skin and epidermal mucus of salmonids. *Toxicology and applied pharmacology*, 44(2):277-289.

65. Schultz, I.R., M.G. Barron, M.C. Newman, and A.M. Vick. 1999. Blood flow distribution and tissue allometry in channel catfish. *Journal of Fish Biology*, 54(6):1275-1286.

66. LaLone, C.A., D.L. Villeneuve, A.W. Olmstead, E.K. Medlock, M.D. Kahl, K.M. Jensen, E.J. Durhan, E.A. Makynen, C.A. Blanksma, and J.E. Cavallin. 2012. Effects of a glucocorticoid receptor agonist, dexamethasone, on fathead minnow reproduction, growth, and development. *Environmental Toxicology and Chemistry*, 31(3):611-622.

67. Le Gac, F., J.L. Thomas, B. Mourot, and M. Loir. 2001. In vivo and in vitro effects of prochloraz and nonylphenol ethoxylates on trout spermatogenesis. *Aquatic toxicology*, 53(3-4):187-200.

68. Baudiffier, D., N. Hinfray, C. Ravaud, N. Creusot, E. Chadili, J.-M. Porcher, R.W. Schulz, and F. Brion. 2013. Effect of in vivo chronic exposure to clotrimazole on zebrafish testis function. *Environmental Science and Pollution Research*, 20(5):2747-2760.

69. Oyelese, O. 2006. Implication of organ and tissue weight to the processing of some selected fresh water fish families. *Journal of fisheries International*, 1(2-4):136-140.

70. Hassanin, A., S. Kuwahara, Nurhidayat, Y. Tsukamoto, K. Ogawa, K. Hiramatsu, and F. Sasaki. 2002. Gonadosomatic Index and Testis Morphology of Common Carp (Cyprinus carpio) in Rivers Contaminated with Estrogenic Chemicals. *Journal of Veterinary Medical Science*, 64(10):921-926.

71. Bagenal, T. 1957. The breeding and fecundity of the long rough dab, Hippoglossoides platessoides (Fabr.) and the associated cycle in condition. *Journal of the Marine Biological Association of the United Kingdom*, 36(2):339-375.

72. Burr, B.M. and R.L. Mayden. 1982. Life history of the brindled madtom Noturus miurus in Mill Creek, Illinois (Pisces: Ictaluridae). *American Midland Naturalist*:25-41.

73. Treasure, J. 1981. Some aspects of the reproductive biology of perch Perca fluviatilis L. Fecundity, maturation and spawning behaviour. *Journal of Fish Biology*, 18(6):729-740.

74. Le Cren, E.D. 1951. The length-weight relationship and seasonal cycle in gonad weight and condition in the perch (Perca fluviatilis). *The Journal of Animal Ecology*:201-219.

75. Garcia, S.N., M. Foster, L.A. Constantine, and D.B. Huggett. 2012. Field and laboratory fish tissue accumulation of the anti-convulsant drug carbamazepine. *Ecotoxicology and Environmental Safety*, 84:207-211.

76. Nkoom, M., G. Lu, J. Liu, and H. Dong. 2020. Biological uptake, depuration and biochemical effects of diclofenac and carbamazepine in Carassius carassius. *Ecotoxicology and Environmental Safety*, 205:111106.

77. Chen, F., Z. Gong, and B.C. Kelly. 2017. Bioaccumulation behavior of pharmaceuticals and personal care products in adult zebrafish (Danio rerio): Influence of physical-chemical properties and biotransformation. *Environmental Science & Technology*, 51(19):11085-11095.

78. Lu, G., Z. Xie, and Z. Zhang. 2018. Effects of dissolved organic matter, feeding, and water flow on the bioconcentration of diclofenac in crucian carp (Carassius auratus). *Environmental Science and Pollution Research*, 25(8):7776-7784.

79. Memmert, U., A. Peither, R. Burri, K. Weber, T. Schmidt, J.P. Sumpter, and A. Hartmann. 2013. Diclofenac: new data on chronic toxicity and bioconcentration in fish. *Environmental Toxicology and Chemistry*, 32(2):442-452.

80. Chen, Q., D. Yin, X. Hu, R. Wang, and C. Zhang. 2014. The effect of nC60 on tissue distribution of ibuprofen in Cyprinus carpio. *Science of the Total Environment*, 496:453-460.

81. Nallani, G.C., P.M. Paulos, L.A. Constantine, B.J. Venables, and D.B. Huggett. 2011. Bioconcentration of ibuprofen in fathead minnow (Pimephales promelas) and channel catfish (Ictalurus punctatus). *Chemosphere*, 84(10):1371-1377.

82. Islas-Flores, H., L.M. Gómez-Oliván, M. Galar-Martínez, S. García-Medina, N. Neri-Cruz, and O. Dublán-García. 2014. Effect of ibuprofen exposure on blood, gill, liver, and brain on common carp (Cyprinus carpio) using oxidative stress biomarkers. *Environmental Science and Pollution Research*, 21(7):5157-5166.

83. Nichols, J.W., B. Du, J.P. Berninger, K.A. Connors, C.K. Chambliss, R.J. Erickson, A.D. Hoffman, and B.W. Brooks. 2015. Observed and modeled effects of pH on bioconcentration of diphenhydramine, a weakly basic pharmaceutical, in fathead minnows. *Environmental Toxicology and Chemistry*, 34(6):1425-1435.

84. Paterson, G. and C.D. Metcalfe. 2008. Uptake and depuration of the anti-depressant fluoxetine by the Japanese medaka (Oryzias latipes). *Chemosphere*, 74(1):125-130.

85. Tanoue, R., K. Nomiyama, H. Nakamura, J.-W. Kim, T. Isobe, R. Shinohara, T. Kunisue, and S. Tanabe. 2015. Uptake and tissue distribution of pharmaceuticals and personal care products in wild fish from treated-wastewater-impacted streams. *Environmental Science & Technology*, 49(19):11649-11658.

86. Fitzsimmons, P.N., J.D. Fernandez, A.D. Hoffman, B.C. Butterworth, and J.W. Nichols. 2001. Branchial elimination of superhydrophobic organic compounds by rainbow trout (Oncorhynchus mykiss). *Aquatic Toxicology*, 55(1):23-34.

87. Schmidt-Nielsen, K. and S.-N. Knut, *Scaling: why is animal size so important?* 1984. Place: Cambridge university press.

88. Davis, J.C. and J.N. Cameron. 1971. Water flow and gas exchange at the gills of rainbow trout, Salmo gairdneri. *Journal of Experimental Biology*, 54(1):1-18.

89. Neumann, P., G.F. Holeton, and N. Heisler. 1983. Cardiac output and regional blood flow in gills and muscles after exhaustive exercise in rainbow trout (Salmo gairdneri). *Journal of Experimental Biology*, 105(1):1-14.

90. Brodeur, J., D. Dixon, and R. McKinly. 2001. Assessment of cardiac output as a predictor of metabolic rate in rainbow trout. *Journal of Fish Biology*, 58(2):439-452.

91. Gamperl, A., A. Pinder, R. Grant, and R. Boutilier. 1994. Influence of hypoxia and adrenaline administration on coronary blood flow and cardiac performance in seawater rainbow trout (Oncorhynchus mykiss). *Journal of Experimental Biology*, 193(1):209-232.

92. Petersen, L., E. Dzialowski, and D. Huggett. 2011. The interactive effects of a gradual temperature decrease and long-term food deprivation on cardiac and hepatic blood flows in rainbow trout (Oncorhynchus mykiss). *Comparative Biochemistry and Physiology Part A: Molecular & Integrative Physiology*, 160(3):311-319.

93. Franklin, C.E., W. Davison, and F. Seebacher. 2007. Antarctic fish can compensate for rising temperatures: thermal acclimation of cardiac performance in Pagothenia borchgrevinki. *Journal of Experimental Biology*, 210(17):3068-3074.

94. Kolok, A.S., M. Spooner, and A.P. Farrell. 1993. The effect of exercise on the cardiac output and blood flow distribution of the largescale sucker Catostomus macrocheilus. *Journal of Experimental Biology*, 183(1):301-321.

95. Cooke, S.J., E.C. Grant, J.F. Schreer, D.P. Philipp, and A.L. Devries. 2003. Low temperature cardiac response to exhaustive exercise in fish with different levels of winter quiescence. *Comparative Biochemistry and Physiology Part A: Molecular & Integrative Physiology*, 134(1):157-165.

96. Taylor, S., S. Egginton, and E. Taylor. 1996. Seasonal temperature acclimatisation of rainbow trout: cardiovascular and morphometric influences on maximal sustainable exercise level. *Journal of Experimental Biology*, 199(4):835-845.

97. Farrell, A., A. Gamperl, J. Hicks, H. Shiels, and K. Jain. 1996. Maximum cardiac performance of rainbow trout (Oncorhynchus mykiss) at temperatures approaching their upper lethal limit. *Journal of Experimental Biology*, 199(3):663-672.

98. Mercier, C., M. Axelsson, N. Imbert, G. Claireaux, C. Lefrancois, J. Altimiras, and A. Farrell. 2002. In vitro cardiac performance in triploid brown trout at two acclimation temperatures. *Journal of fish biology*, 60(1):117-133.

99. Keen, J. and A. Farrell. 1994. Maximum prolonged swimming speed and maximum cardiac performance of rainbow trout, Oncorhynchus mykiss, acclimated to two different water

temperatures. *Comparative Biochemistry and Physiology Part A: Physiology*, 108(2-3):287-295.

100. Wang, T., S. Lefevre, N.K. Iversen, I. Findorf, R. Buchanan, and D.J. McKenzie. 2014. Anaemia only causes a small reduction in the upper critical temperature of sea bass: is oxygen delivery the limiting factor for tolerance of acute warming in fishes? *Journal of Experimental Biology*, 217(24):4275-4278.

101. Schreer, J.F., S.J. Cooke, and R. McKinley. 2001. Cardiac response to variable forced exercise at different temperatures: an angling simulation for smallmouth bass. *Transactions of the American Fisheries Society*, 130(5):783-795.

102. Clark, T.D., E. Sandblom, G.K. Cox, S.G. Hinch, and A.P. Farrell. 2008. Circulatory limits to oxygen supply during an acute temperature increase in the Chinook salmon (Oncorhynchus tshawytscha). *American Journal of Physiology-Regulatory, Integrative and Comparative Physiology*, 295(5):R1631-R1639.

103. Brijs, J., E. Sandblom, E. Dekens, J. Näslund, A. Ekström, and M. Axelsson. 2017. Cardiac remodeling and increased central venous pressure underlie elevated stroke volume and cardiac output of seawater-acclimated rainbow trout. *American Journal of Physiology-Regulatory, Integrative and Comparative Physiology*, 312(1):R31-R39.

104. Blank, J.M., J.M. Morrissette, P.S. Davie, and B.A. Block. 2002. Effects of temperature, epinephrine and Ca2+ on the hearts of yellowfin tuna (Thunnus albacares). *Journal of Experimental Biology*, 205(13):1881-1888.

105. Mendonça, P.C. and A.K. Gamperl. 2010. The effects of acute changes in temperature and oxygen availability on cardiac performance in winter flounder (Pseudopleuronectes americanus). *Comparative Biochemistry and Physiology Part A: Molecular & Integrative Physiology*, 155(2):245-252.

106. Butler, P. and E. Taylor. 1975. The effect of progressive hypoxia on respiration in the dogfish (Scyliorhinus canicula) at different seasonal temperatures. *Journal of Experimental Biology*, 63(1):117-130.

107. Joyce, W., M. Axelsson, S. Egginton, A.P. Farrell, E.L. Crockett, and K.M. O'Brien. 2018. The effects of thermal acclimation on cardio-respiratory performance in an Antarctic fish (Notothenia coriiceps). *Conservation physiology*, 6(1):coy069.

108. Joaquim, N., G.N. Wagner, and A.K. Gamperl. 2004. Cardiac function and critical swimming speed of the winter flounder (Pleuronectes americanus) at two temperatures. *Comparative Biochemistry and Physiology Part A: Molecular & Integrative Physiology*, 138(3):277-285.

109. Gollock, M., S. Currie, L. Petersen, and A. Gamperl. 2006. Cardiovascular and haematological responses of Atlantic cod (Gadus morhua) to acute temperature increase. *Journal of Experimental Biology*, 209(15):2961-2970.

110. Sandblom, E., T.D. Clark, A. Gräns, A. Ekström, J. Brijs, L.F. Sundström, A. Odelström, A. Adill, T. Aho, and F. Jutfelt. 2016. Physiological constraints to climate warming in fish follow principles of plastic floors and concrete ceilings. *Nature Communications*, 7(1):1-8.

111. Steinhausen, M., E. Sandblom, E. Eliason, C. Verhille, and A. Farrell. 2008. The effect of acute temperature increases on the cardiorespiratory performance of resting and swimming sockeye salmon (Oncorhynchus nerka). *Journal of Experimental Biology*, 211(24):3915-3926.

112. Benson, B.B. and D. Krause Jr. 1980. The concentration and isotopic fractionation of gases dissolved in freshwater in equilibrium with the atmosphere. 1. Oxygen. *Limnology and Oceanography*, 25(4):662-671.

113. Stadnicka, J., K. Schirmer, and R. Ashauer. 2012. Predicting concentrations of organic chemicals in fish by using toxicokinetic models. *Environmental Science & Technology*, 46(6):3273-3280.
